# Constructing Dynamical Symmetries for Quantum Computing: Applications to Coherent Dynamics in Coupled Quantum Dots

**DOI:** 10.3390/nano14242056

**Published:** 2024-12-23

**Authors:** James R. Hamilton, Raphael D. Levine, Francoise Remacle

**Affiliations:** 1 Theoretical Physical Chemistry, UR MOLSYS, University of Liege, B4000 Liège, Belgium; jamesross.hamilton@mail.huji.ac.il; 2 Institute of Chemistry, The Hebrew University of Jerusalem, Jerusalem 91904, Israel; raphy@mail.huji.ac.il; 3 Department of Molecular and Medical Pharmacology, David Geffen School of Medicine, University of California Los Angeles, Los Angeles, CA 90095, USA; 4 Department of Chemistry and Biochemistry, University of California Los Angeles, Los Angeles, CA 90095, USA

**Keywords:** Lie algebra, coherent quantum dynamics, computing by observables, CdSe nanoparticle dimers

## Abstract

Dynamical symmetries, time-dependent operators that almost commute with the Hamiltonian, extend the role of ordinary symmetries. Motivated by progress in quantum technologies, we illustrate a practical algebraic approach to computing such time-dependent operators. Explicitly we expand them as a linear combination of time-independent operators with time-dependent coefficients. There are possible applications to the dynamics of systems of coupled coherent two-state systems, such as qubits, pumped by optical excitation and other addressing inputs. Thereby, the interaction of the system with the excitation is bilinear in the coherence between the two states and in the strength of the time-dependent excitation. The total Hamiltonian is a sum of such bilinear terms and of terms linear in the populations. The terms in the Hamiltonian form a basis for Lie algebra, which can be represented as coupled individual two-state systems, each using the population and the coherence between two states. Using the factorization approach of Wei and Norman, we construct a unitary quantum mechanical evolution operator that is a factored contribution of individual two-state systems. By that one can accurately propagate both the wave function and the density matrix with special relevance to quantum computing based on qubit architecture. Explicit examples are derived for the electronic dynamics in coupled semi-conducting nanoparticles that can be used as hardware for quantum technologies.

## 1. Introduction

The dual role of operators that commute with the Hamiltonian as symmetries and as constants of the motion was established very early in quantum mechanics. The application of symmetry was developed in detail as ‘group theory’, and it became a central component in the bag of tools of chemists, see, e.g., [1]. It was only in the sixties of the previous century that the notion of symmetry was extended to groups of operators that do not necessarily commute with the Hamiltonian, see, for example, the seminal paper of Lewis and Riesenfeld [2]. As far as we know, there were at least three lines of independent developments at the time. In retrospect, these developments are closely related. The first development is primarily of a mathematical nature. It is to seek analytical solutions of exponential forms of linear differential equations of the first order. The time-dependent Schrödinger equation for the wave function is an equation of this type, as are other well-known equations of mathematical physics (e.g., the diffusion equation, the master equation). Among these equations, the Schrödinger equation is almost unique in that it describes reversible dynamics. A rigorous exponential type solution was presented by Magnus [3]. An early application of this work in physicochemical dynamics is by Pechukas and Light [4], and a detailed review of the earlier work is by Wilcox [5]. An early application in optics was by Hioe and Eberly [6]. See also [7], Dattoli [8,9,10,11], and Altafini [12,13]. Below, we use a complementary early mathematical representation of the exponential by Norman and Wei [14,15]. The essential difference with the earlier work is that we will seek methods that can also be used to describe and solve the time evolution of the quantum mechanical density matrix, a quantity that is bilinear in the quantum amplitudes [16]. Beginning in pure mathematics, there was also a more general approach that sought to identify symmetries of more general differential equations [17,18]. See also the papers [9,19,20,21,22,23,24]. The work of Wulfman, with special reference to time dilation, as summarized in his book [25], is perhaps the best known application in chemical physics. The second development was motivated by the physics of elementary particles. The notion of a dynamical symmetry was introduced there, and a detailed review is by Bohm, Ne’eman, and Barut [26]. An early application of this concept in scattering theory is by Alhassid and Levine [27], see also [28]. Early overviews are [29,30,31]. It was also shown [32] that by elevating time to the role of a dynamical variable, the dynamical symmetries become stationary constants of the motion. The third development was motivated by extending the notion of a coherent state as discussed in the books [33,34,35]. See also [36,37,38,39,40].

Our intention in this paper is to report on the actual explicit construction of dynamical symmetries using a variant of the procedure of Wei and Norman [14,15]. We start in Section 2 by defining dynamical symmetries. Then, in Section 3, we outline our presentation of the Wei–Norman approach. This provides an explicit, product form for the evolution operator, and this operator can then be applied to any initial quantum wave function. We then use the product form of the evolution operator to propagate operators of interest. Our approach differs in an essential manner from the derivation of Alhassid and Levine [28]. Their derivation is simpler at the important price that it applies only to the particular initial state of interest. On the other hand, the derivation in Section 3, a derivation based on the product form of the evolution operator, applies to a general initial state.

The construction of an evolution operator can be used to propagate the wave function in time. But our intention is to propagate, in time, the density matrix, a bilinear quantity that lives in Liouville space [16,41] rather than the wave function that lives in Hilbert space. We suggest that this is not only an alternative approach but a method that brings dynamical symmetries to the forefront. A computation is done by a physical system that changes its state by an application of an external input. The density matrix is a most suitable framework to describe this. The key point, a point that will be elaborated in the technical discussion, is that the density matrix itself is a dynamical symmetry (in classical mechanics this is often stated as the Liouville theorem). The dynamical symmetries are therefore an optimal basis of operators for expressing the density matrix. In this paper we deal specifically with systems of coupled qubits (that is, coupled two-level systems) but our method is a general approach that has other potential applications. Explicit results for a two-level system and the generalization to a *N*-level system are discussed in Section 4. The factorization of the evolution operator is both interesting and potentially practical. Our longer range motivation is quantum technologies, and so, we make the obvious statement that a two-level system is a qubit and proceed in Section 5 to show that the *N*-level system can be discussed as *N*(*N*−1)/2 coupled qubits, see Section 3 below. We illustrate the advantages of our approach by computing the electronic dynamics for models of semi-conducting single, small, 3-nm in diameter, colloidal CdSe nanoparticles and nanoparticle dimers, which are optically addressed [42]. Devices based on semi-conducting nanoparticles are used in a wide range of applications in nanotechnologies [23,43,44,45,46,47,48]. Algorithm implementations based on spin in solid-state quantum dot hardware have been proposed since the early days of quantum computing [49,50,51,52,53]. We showed that colloidal CdSe nanoparticles can be assembled in multilayered devices, operate at room temperature [54,55], and provide a suitable hardware for implementing quantum algorithms [56,57].

## 2. Dynamical Symmetries

Dynamical symmetries, $A_{r}(t)$, are operators defined as

(1) $$idA_{r}(t)/dt=i\partial A_{r}(t)/\partial t-\left[H,A_{r}(t)\right]=0$$

with the initial condition, $A_{r}\left(0\right)=A_{r}$, where $A_{r}$ is an operator in the usual Schrödinger picture. We use boldface symbols for operators because we aim for a practical construction, which means that we will work in a finite dimensional Hilbert space where an operator is a matrix.

By comparing Equation (1) to the Heisenberg equation of motion for an operator, one can say that since the unitary time evolution operator $U\left(t\right)$ satisfies $U^{†}\left(-t\right)=U\left(t\right),$ the dynamical symmetries are Heisenberg picture operators that move backwards in time,

(2) $$A_{r}\left(t\right)=U\left(t\right)A_{r}U^{†}\left(t\right)$$

we use the usual boundary conditions that $U\left(t\right)=I$.

The aim of this paper is to construct a form of the evolution operator of the Hamiltonian H that is particularly useful for computing the dynamical symmetries starting with the formal solution, Equation (2), particularly so when given a set of operators $\left\{A_{r}\right\},\;r=1,\ldots ,n$ that forms a Lie algebra meaning that the set is closed under commutation, $\left[A_{r},\;A_{s}\right]=\sum_{t=1}^{n}C_{rs}^{t}A_{t}$. In the mathematical literature mentioned in the introduction, it is typically assumed that the generator of the time evolution that, for us here, is the Hamiltonian H itself, is in algebra and that it can be time dependent in form

(3) $$H=\sum_{r}h_{r}(t)A_{r}$$

The operators $\left\{A_{r}\right\}$ are members of Lie algebra and the time-dependent coefficients are real or complex as needed so that the Hamiltonian is Hermitian. For this special form of the Hamiltonian, the dynamical symmetries can be expressed as linear combinations of the operators of algebra

(4) $$A_{r}(t)=\sum_{s}a_{rs}(t)A_{s}$$

A central aim of this paper is to determine the time-dependent coefficients $a_{rs}(t)$ in a systematic and realistic manner valid for any operator $A_{r}\left(0\right)=A_{r}$. In principle, one can substitute Equation (4) for $A_{r}(t)$ as an ansatz in Equation (1) that defines the dynamical symmetries. This will provide a set of coupled equations of motion for the time dependence of the set of coefficients $a_{rs}(t)$ that can be solved for a particular set of initial conditions $a_{rs}(0)$. Our aim here is to find a general solution that we do, as we now discuss, by first solving for the evolution operator, see Equation (2). Thereby, we have a general scheme.

We can equally regard the dynamical symmetries as operators in the Schrödinger picture. The Heisenberg picture operators that correspond to the Schrödinger picture are $U^{†}\left(t\right)A_{r}\left(t\right)U\left(t\right)=A_{r}$ thereby providing a clear indication that the dynamical symmetries are constant of the motion. To a mathematician this follows from the result that they commute with H−i∂/∂t.

A proof in terms of expectation values starts with density operator $\rho \left(t\right)$, which describes the system at time *t*. $\rho \left(t\right)$ evolves in time according to the well-known Liouville–von Neumann equation, $i\partial \rho /\partial t=\left[H,\rho \right]$, where H is the Hamiltonian, and we took ℏ=1. The formal solution is a unitary time evolution $\rho \left(t\right)=U\left(t\right)\rho \left(0\right)U^{†}\left(t\right).$ It then follows that the expectation value of a dynamical symmetry is conserved

(5) $$Tr\left(\rho \left(t\right)A_{r}\left(t\right)\right)=Tr(U\left(t\right)\rho \left(0\right)U^{†}\left(t\right)U\left(t\right)A_{r}U^{†}\left(t\right))=Tr(\rho \left(0\right)A_{r})$$

In this paper, we take it that a practical procedure is typically limited to a Hilbert space that is of a finite dimension, *N*. The dimension can be large but finite. It follows that, for our purpose, an operator can be represented as an N by N matrix, and this is why we use boldface symbols. A suitable basis of operators are the $N^{2}$ Hermitian operators, {|i⟩⟨j|}, where |i⟩, |j⟩ for i,j∈{1,…N}, are linearly independent N basis states in the Hilbert space. Then the diagonal elements, the populations, are specified by the mean values of the N states |i⟩⟨i|. The coherences are specified by the $N\left(N-1\right)$ complex numbers ⟨|i⟩⟨j|⟩, which are pairwise conjugates to one another, that is by $N\left(N-1\right)/2$ real numbers. These $N^{2}$ operators are the generators of Lie algebra U(N). In an advanced text, they will be called the Cartan–Weyl basis for that algebra. If we separately require that the populations are normalized, then we need a total of only additional $N^{2}-1$ basis operators and the algebra will be SU(N).

One can be concerned that the assumption about the linear structure of the Hamiltonian, Equation (3), is too restrictive. Indeed, in many body problems in physics and chemistry and particularly in highly correlated systems, it is convenient to start with a Hamiltonian that is bilinear in the physically motivated observables. It is then natural to approximate the solution using a mean field approximation to linearize the Hamiltonian. Others and we [40,58,59,60] have discussed and demonstrated how an effective approximate linearization can be achieved. Here we proceed in a different way by pointing out a reality of our digital age. In many realistic applications, we will work in an enumerable, N dimensional, Hilbert space. Then, there is a Cartan–Weyl type basis for U(N), as discussed above. It can well be that smaller algebra is enough, but with $N^{2}$ operators, we can take a system of N states that are pairwise coupled and write the Hamiltonian as a linear sum over $N^{2}$ terms, $H=\sum_{i,j}H_{ij}\left|i\right\rangle \left\langle j\right|.$ This is an idea that goes back to Dirac [61], where each basis quantum state is shown to be mathematically analogous to a classical harmonic oscillator. One can, if it proves useful, also consider such a Hamiltonian as being of an Ising type. This requires that one thinks of each ‘spin’ asa state, and these states are pairwise coupled. Another useful relation is to write the Ising Hamiltonian as $H=\sum_{i}\sum_{j>i}(H_{ii}\left|i\right\rangle \left\langle i\right|+H_{jj}\left|j\right\rangle \left\langle j\right|+H_{ij}(\left|i\right\rangle \left\langle j\right|+\left|j\right\rangle \left\langle i\right|$, which shows that the Ising Hamiltonian can be rewritten as a sum of $N\left(N-1\right)/2$ coupled two-state systems. This is our direct connection to quantum computers constructed as coupled qubits.

## 3. The Evolution Operator in a Product Form

Our aim is to determine explicitly the dynamical symmetries as an explicit expression in terms of the time independent closed set of operators $\left\{A_{r}\right\}$. To do so, we need to propagate these operators in time, actually backwards in time. Previously [28,30], we directly solved the Heisenberg equation of motion, Equation (1), for the set of operators that are relevant in the system of interest. Here, we aim to allow for a more general initial state. To move in time for any quantum mechanical state, we need the evolution operator. For the given closed set of operators and when the Hamiltonian is a linear expression of members of the set, Equation (3), we follow the construction of Wei and Norman [14,15] to obtain the evolution operator for the Hamiltonian. We caution already very early that while we use the approach of Wei and Norman to determine the evolution operator, the time correlation matrix that we are after is different from the time correlation matrix of Wei and Norman. Both matrices are tightly defined, and there is a good reason why they are quite different. The matrix we require propagates operators backwards in time under the action of the full Hamiltonian of the system. We need to propagate in Liouville space. The correlation matrix of Wei and Norman propagates wave functions in Hilbert space.

The starting technical development is the parametrization of the time evolution operator in a product form as proposed by Wei and Norman [14,15]

(6) $$U(t)=exp\left(g_{1}\left(t\right)X_{1}\right)exp\left(g_{2}\left(t\right)X_{2}\right)\ldots exp(g_{\nu }\left(t\right)X_{\nu })$$

where ν is the number of generators of the algebra and the *g*’s are functions of time that needs to be determined. From here on, we use the notation $X_{k}$ to denote generators that are skew-Hermitian operators, i.e., where the $\left\{-iX_{k}\right\}$ are Hermitian. With this condition the evolution operator U(t) of Equation (6) is unitary when the $\left\{g_{k}\left(t\right)\right\}$ are real.

The *N*-state system unitary evolution operator U is comprised of ν different factors $exp(g_{k}X_{k})$. The factors can be grouped into sets of three, each constituting an SU(2) group. There are η groups with ν=3η.

The three skew-Hermitian generators of each group are taken to involve two quantum states. Labelling the two quantum states *i* and *j,* the three generators of each SU(2) subgroup have the form

(7) $$X_{a}=i\left(E_{ij}+E_{ji}\right);X_{b}=\left(E_{ij}-E_{ji}\right);{\;X}_{c}=i\left(E_{ii}-E_{jj}\right)$$

where $E_{ij}=\left|i\right\rangle \left\langle j\right|,$ is the coherence or, for i=j, the population observable. Sometimes the $\left\{E_{ij}\right\}$ are called Gelfand operators.

In the following, we very briefly sketch the factorization approach for one SU(2) group using the generators as shown in Equation (7). A more detailed discussion is provided in the Supplementary Materials, Section S1.2 and also in the ref. [62]. We then construct the factorization of the evolution operator and the construction of the dynamical symmetries for a 3-state system described by three coupled SU(2) algebras, based upon a generalization of the construction for an *N* state system. See also the work of Hioe and Eberly on three coupled states [63].

Equation (7) is not the most common basis for SU(2). However, it is a basis previously used to advantage by Altafini [12,13] and in ref. [62], and it proves convenient for our purpose of computing the group parameters $\left\{g_{k}\right\}$ that, for the skew Hermitian operators X are then real for a unitary U.

This approach also has the advantage of providing a direct generalization for *N* state systems. For a system of N quantum states, there will be $\eta =N\left(N-1\right)/2$ distinct pairs of states, so η is therefore the number of coupled SU(2) algebra. There are three generators X per each SU(2), so the total number of generators is ν=3η. The values of ν and η are given in Table 1 for different values of N.

The dependence of the evolution operator in the product form, Equation (6), on the set of parameters $\left\{g_{k}\right\}$ is [14]

(8) $$\partial U/\partial g_{k}=\left(\prod_{j=1}^{k-1}exp\left(g_{j}X_{j}\right)\right)X_{k}\left(\prod_{j=k}^{\nu }exp\left(g_{j}X_{j}\right)\right)$$

To write this in a more compact form Wei and Norman define a matrix Ξ with, the elements ξ of which are defined as

(9) $$\;(\partial U/\partial g_{k})U^{-1}=\left(\prod_{j=1}^{k-1}exp\left(g_{j}X_{j}\right)\right)X_{k}\left(\prod_{j=k-1}^{1}exp\left(-g_{j}X_{j}\right)\right)\equiv \sum_{m=1}^{\nu }\xi_{mk}X_{m}$$

Here, the matrix elements $\xi_{mk}$ depend on ($\nu -1)\;g_{k}’s$, $\xi_{mk}\left(g_{1},g_{2},\ldots ,g_{\nu -1}\right)$. As defined, *m* is an index of a row of the Ξ matrix while *k* is an index of a column. See the Supplementary Sections S1.1 and S2.1 for a full enumeration of the 2- and 3-state Ξ’s, respectively, in the skew-Hermitian basis defined by Equation (7). The matrix elements $\xi_{mk}$, through the $\left\{g_{k}(t)\right\}$, are functions of time, $\xi_{mk}\left(g_{1}\left(t\right),g_{2}\left(t\right),\ldots ,g_{\nu -1}\left(t\right)\right)$. As a function of $\left\{g_{k}\right\}$, Ξ can therefore be determined from the commutation relations of the algebra without reference to any particular Hamiltonian. The correlation matrix $\Xi \left(g_{1},g_{2},\ldots ,g_{\nu -1}\right)$ is an invertible, non-symmetric ν by ν matrix. Its specific form depends on the form of the operators used to close the algebra and of their order in Equation (6). Using a notation of matrix algebra, an alternative form of the *k*th column of the Ξ matrix is $exp(g_{1}adX_{1})exp(g_{2}adX_{2})...exp(g_{k-1}adX_{k-1})\;X_{k}$, where the operation $adX_{m}$ on an operator $X_{k}$ is defined as $\left(adX_{m}\right)X_{k}=\left[X_{m},X_{k}\right]$, so that ${\left(adX_{m}\right)}^{2}X_{k}=\left[X_{m},\left[X_{m},X_{k}\right]\right],$ etc.

The equations of motion for the $\left\{g_{k}\left(t\right)\right\}$ are derived by differentiating (6) wrt time. This is described in detail by Wei and Norman and, in our notation, in Section S1 of the Supplementary Materials

We write the matrix elements $\xi_{mk}$ as functions of the $\left\{g_{k}\right\}$ because they are determined by the algebra for all the possibly time-dependent Hamiltonians that are linear functions of the generators. The matrix Ξ is a real analytic function with an initial value of I at t=0. We have explicit ν equations of motion separately for each one of the ν parameters of the evolution operator

(10) $$dg_{m}/dt=\sum_{k}{\left(\Xi^{-1}\right)}_{mk}h_{k}\left(t\right),\;m=1,\ldots ,\nu$$

We reiterate that the matrix Ξ is a function of the $\left\{g_{k}\right\}$. So, the equations of motion are first order in time but they are not linear equations, and they are coupled. The initial values for all are $g_{k}\left(t=0\right)=0$, so that there is an explicit solution at least for short times.

The next step is to extend the definition of the matrix elements, $\xi_{mk}$, Equation (9), for all ν sets of parameters, r=1,2,…,ν

(11) $$\left(\prod_{j=1}^{r}exp\left(g_{j}X_{j}\right)\right)X_{k}\left(\prod_{j=r}^{1}exp\left(-g_{j}X_{j}\right)\right)\equiv \sum_{m=1}^{\nu }\xi_{mk}X_{m}\;\forall \;r=1,2,\ldots ,\nu \;\xi_{mk}=\xi_{mk}\left(g_{1},g_{2},\ldots ,g_{r}\right)$$

For the case r=ν, this expresses the full dynamical symmetry $X_{k}\left(t\right)=U\left(t\right)X_{k}U^{†}(t)$ as a linear combination of time independent, Schrödinger picture, operators, Equation (12)

(12) $$X_{k}\left(t\right)=\sum_{j=1}^{\upsilon }a_{kj}\left(t\right)X_{j}$$

We next intend to demonstrate a computation of the dynamical symmetries as coupled SU(2) algebras, where each algebra is a coherent two-level system and so, a qubit. We present analytical results for one qubit system and for three coupled qubits, as well as the generalization for the N(N−1)/2 coupled qubits that can be built for a *N* state system. Our tool, as already hinted earlier, is to write the Hamiltonian as a linear combination of the diagonal operators |i⟩⟨i| and the off diagonal ones |i⟩⟨j|, where the $\left\{\left|i\right\rangle \right\}$ are the basis states, typically these are the eigenstates of the Hamiltonian in the absence of input so that, without input, the state of the system is stationary.

## 4. Explicit Solution of the Two-Coupled Level System

For the coupled qubits problem and to be consistent with the notation in Lie algebraic papers, we use $X_{k}$’s for the Schrödinger operators with $E_{ij}=\left|i\right\rangle \left\langle j\right|$. For a two-level system

(13) $$I=E_{11}+E_{22}$$

(14) $$X_{1}=\left(\begin{array}{cc} 0 & i\\ i & 0 \end{array}\right)=i\left(E_{12}+E_{21}\right)$$

(15) $$X_{2}=\left(\begin{array}{cc} 0 & 1\\ -1 & 0 \end{array}\right)=\left(E_{12}-E_{21}\right)$$

(16) $$X_{3}=\left(\begin{array}{cc} i & 0\\ 0 & -i \end{array}\right)=i\left(E_{11}-E_{22}\right)$$

This is almost the same basis as was used by Altafini [12,13] and in ref. [62]. The input is provided by a time dependent optical pulse E(t), so that the full Hamiltonian operator

(17) $$H\left(t\right)=0E_{11}-E\left(t\right)\mu E_{12}-E(t)\mu E_{21}+\alpha E_{22}$$

where μ is the transition dipole moment. In matrix form, in the two-dimensional Hilbert space,

(18) $$H(t)=\left(\begin{array}{cc} 0 & -E\left(t\right)\mu \\ -E\left(t\right)\mu & \alpha \end{array}\right)$$

The ground state is taken to be at energy zero, so that α is the energy of excitation. Equation (18) can be rewritten in terms of the SU(2) operators

(19) $$H=iE\left(t\right)\mu X_{1}+i\frac{\alpha }{2}X_{3}+\frac{\alpha }{2}I$$

so that the vector of the coefficients of the operators in the Hamiltonian, $h\left(t\right)$ (see Equation (3)) is

(20) $$h^{T}\left(t\right)=\left(\begin{array}{ccc} iE\left(t\right)\mu & 0 & \alpha /2 \end{array}\right)$$

The commutation relations of the SU(2) operators in the form that we use are Table S1.

The evolution operator is chosen to be in a sequential order of operators

(21) $$U(t)=exp(g_{1}\left(t\right)X_{1})exp(g_{2}\left(t\right)X_{2})exp(g_{3}\left(t\right)X_{3})$$

where for our choice of skew-Hermitian operators $\left\{X_{k}\right\}$, we will need to verify that the results for the $\left\{g_{k}\right\}$ are real in order that the evolution operator is unitary.

Using the commutation of the SU(2) operators, we compute the elements of the Ξ matrix that governs the time evolution of the $\left\{g_{k}\right\}$ (Equation (10)).

The Ξ matrix, as solved in the Supplementary Materials, and its inverse are

(22) $$\Xi =\left(\begin{array}{ccc} 1 & 0 & -\sin\left(2g_{2}\right)\\ 0 & \cos\left(2g_{1}\right) & \cos\left(2g_{2}\right)\sin\left(2g_{1}\right)\\ 0 & -\sin\left(2g_{1}\right) & \cos\left(2g_{2}\right)\cos\left(2g_{1}\right) \end{array}\right),\;\Xi^{-1}=\left(\begin{array}{ccc} 1 & \tan\left(2g_{2}\right)\sin\left(2g_{1}\right) & \tan\left(2g_{2}\right)\cos\left(2g_{1}\right)\\ 0 & \cos\left(2g_{1}\right) & -\sin\left(2g_{1}\right)\\ 0 & \sec\left(2g_{2}\right)\sin\left(2g_{1}\right) & \sec\left(2g_{2}\right)\cos\left(2g_{1}\right) \end{array}\right)$$

and from $\dot{g}=-i\Xi^{-1}h\left(t\right)$ (Equation (10)) we get three coupled differential equations for the $\left\{g_{k}\left(t\right)\right\}$ where the overdot denotes a time derivative

(23) $$\dot{g_{1}}=E\left(t\right)\mu +\frac{\alpha }{2}\tan\left(2g_{2}\right)\cos\left(2g_{1}\right)\;\dot{g_{2}}=-\frac{\alpha }{2}\sin\left(2g_{1}\right)\;\dot{g_{3}}=\frac{\alpha }{2}\sec\left(2g_{2}\right)\cos\left(2g_{1}\right)$$

where as stated below Equation (10), $g_{1}\left(0\right),=g_{2}\left(0\right)=g_{3}\left(0\right)=0.$ After solving these coupled differential equations for the $\left\{g_{k}\left(t\right)\right\}$ as a function of time we have an explicit form of the evolution operator, $U\left(t\right)$, as a product of three exponential terms. Each such term can be represented in Hilbert space as a two by two matrix. Multiplying the three matrices, we get a matrix representation for the evolution operator of a two-state system, see Supplementary Section S1.2,

(24) $$\begin{array}{r} U=\\ \left(\begin{array}{cc} e^{ig_{3}}\left(cos\left(g_{1}\right)cos\left(g_{2}\right)-i\;sin\left(g_{1}\right)\;sin\left(g_{2}\right)\right) & e^{-ig_{3}}\left(cos\left(g_{1}\right)\;sin\left(g_{2}\right)+i\;sin\left(g_{1}\right)\;cos\left(g_{2}\right)\right)\\ -e^{ig_{3}}\left(cos\left(g_{1}\right)\;sin\left(g_{2}\right)-i\;sin\left(g_{1}\right)\;cos\left(g_{2}\right)\right) & e^{-ig_{3}}\left(cos\left(g_{1}\right)\;cos\left(g_{2}\right)+i\;sin\left(g_{1}\right)\;sin\left(g_{2}\right)\right) \end{array}\right) \end{array}$$

To compute the dynamical symmetries, and because U is unitary, we also need its inverse

(25) $$\begin{array}{r} U^{-1}=\\ \left(\begin{array}{cc} e^{-ig_{3}}\left(cos\left(g_{1}\right)cos\left(g_{2}\right)+isin\left(g_{1}\right)sin\left(g_{2}\right)\right) & -e^{-ig_{3}}\left(cos\left(g_{1}\right)sin\left(g_{2}\right)+isin\left(g_{1}\right)cos\left(g_{2}\right)\right)\\ e^{ig_{3}}\left(cos\left(g_{1}\right)sin\left(g_{2}\right)-isin\left(g_{1}\right)cos\left(g_{2}\right)\right) & e^{ig_{3}}\left(cos\left(g_{1}\right)cos\left(g_{2}\right)-isin\left(g_{1}\right)sin\left(g_{2}\right)\right) \end{array}\right) \end{array}$$

Lastly, we bring from the Supplementary the form of the dynamical symmetry operators of SU(2) as $X_{k}(t)=UX_{k}U^{-1}$.

(26) $$\begin{array}{r} X_{1}(t)=cos\left(2g_{2}\right)\;cos\left(2g_{3}\right)\;X_{1}+\left(sin\left({2g}_{1}\right)\;sin\left(2g_{2}\right)\;cos\left(2g_{3}\right)-cos\left(2g_{1}\right)\;sin\left(2g_{3}\right)\right){\;X}_{2}\\ +\left(cos\left({2g}_{1}\right)\;sin\left(2g_{2}\right)\;cos\left(2g_{3}\right)+sin\left(2g_{1}\right)\;sin\left(2g_{3}\right)\right){\;X}_{3}\\ X_{2}(t)=cos\left(2g_{2}\right)\;sin\left(2g_{3}\right)\;X_{1}+\left(cos\left({2g}_{1}\right)\;cos\left(2g_{3}\right)+sin\left(2g_{1}\right)\;sin\left(2g_{2}\right)\;sin\left(2g_{3}\right)\right){\;X}_{2}\\ +\left(cos\left({2g}_{1}\right)\;sin\left(2g_{2}\right)\;sin\left(2g_{3}\right)-sin\left(2g_{1}\right)\;cos\left(2g_{3}\right)\right)\;X_{3}\\ X_{3}(t)=-sin\left(2g_{2}\right){\;X}_{1}+sin\left(2g_{1}\right)\;cos\left(2g_{2}\right)\;X_{2}+cos\left(2g_{1}\right)cos\left(2g_{2}\right){\;X}_{3} \end{array}$$

the time correlation matrix from the Schrödinger picture to the dynamical symmetries, Equation (12), is derived in the Supplementary, Section S1.4. The $X_{k}$’s are Heisenberg operators that move backwards in time, and one verifies that the two time correlation matrices are indeed inverse to one another. For more detailed results of the two-state system, see Section S1 of the Supplementary.

Using the evolution operator, one can propagate in time any initial state that can be specified by the three generators, $\rho \left(t\right)=U\left(t\right)\rho \left(t=0\right)U^{-1}\left(t\right)$. When at time t=0, the system is in its ground state, and we have

$$\begin{array}{r} \rho \left(t\right)=U\left(t\right)\left(\begin{array}{cc} 1 & 0\\ 0 & 0 \end{array}\right)U^{-1}\left(t\right)=exp\left(U\left(t\right)\left(\frac{1}{2}\left(I-iX_{3}\right)\right)U^{-1}\left(t\right)\right)\\ =exp\left(\frac{1}{2}\left(I-iX_{3}\left(t\right)\right)\right)=\left(\begin{array}{cc} \rho_{11} & \rho_{12}\\ \rho_{21} & \rho_{22} \end{array}\right) \end{array}$$

where

(27) $$\begin{array}{r} \rho_{11}\left(t\right)={cos}^{2}\left(g_{1}\right)\;{cos}^{2}\left(g_{2}\right)+{sin}^{2}\left(g_{1}\right)\;{sin}^{2}\left(g_{2}\right)\\ \rho_{12}\left(t\right)=-\left(cos\left(g_{1}\right)\;sin\left(g_{2}\right)+i\;sin\left(g_{1}\right)\;cos\left(g_{2}\right)\right)\left(cos\left(g_{1}\right)cos\left(g_{2}\right)-i\;sin\left(g_{1}\right)\sin\left(g_{2}\right)\right)\\ \rho_{21}(t)=i\;\left(sin\left(g_{1}\right)cos\left(g_{2}\right)+i\;cos\left(g_{1}\right)sin\left(g_{2}\right)\right)\left(cos\left(g_{1}\right)cos\left(g_{2}\right)+i\;sin\left(g_{1}\right)\;sin(g_{2})\right)\\ \rho_{22}(t)={sin}^{2}\left(g_{1}\right)\;{cos}^{2}\left(g_{2}\right)+{cos}^{2}(g_{1}){\;sin}^{2}(g_{2}) \end{array}$$

The final matrix form is an explicit result and shows that the elements of the density matrix of exponential form in a dynamical symmetry are not necessarily simple exponentials.

Matrix multiplication explicitly verifies that the expectation values of the dynamical symmetries for the density matrix at time *t* are time independent and equal to the initial values of the generators (that are 0, 0, and *i*, respectively—see Section S1.4 of the Supplementary Material).

## 5. Generalization to a N-Coupled Level System

The *N*-level unitary evolution operator is given in product form in Equation (6) above. For *N* levels, we have η=N(N−1)/2 pairs of states. Each pair of states corresponds to a qubit and is described by three skewed-Hermitian generators, constituting $SU\left(2\right)$ algebra, Equation (7). The η $SU\left(2\right)$ algebras are coupled because they have states in common. We have a set of ν=3η operators (Table 1), 2η generators for the coherences, and η generators for the population differences. This set of ν=3η generators is closed under commutation. We give in this section the generalizations of the approach outlined for two levels in Section 4 to *N* levels. Examples of a three-level and nine-level model are presented in Section 6.

Using Equation (7) above, for a three-level system, we have three pairs of states 1,2, 1,3, and 2,3, each described by $SU\left(2\right)$ algebra, which leads to nine generators

$$X_{a}=i\left(E_{12}+E_{21}\right);X_{b}=\left(E_{12}-E_{21}\right);X_{c}=i\left(E_{11}-E_{22}\right)$$

$$Y_{a}=i\left(E_{13}+E_{31}\right);Y_{b}=\left(E_{13}-E_{31}\right);Y_{c}=i\left(E_{11}-E_{33}\right)$$

$$Z_{a}=i\left(E_{23}+E_{32}\right);Z_{b}=\left(E_{23}-E_{32}\right);Z_{c}=i\left(E_{22}-E_{33}\right)$$

The operators of each $SU\left(2\right)$ group obey the commutation relations, i.e., for states 1,2

(28) $$\left[X_{a},X_{b}\right]=-2X_{c};\;\left[X_{a},X_{c}\right]=2X_{b};\;\left[X_{b},X_{c}\right]=-2X_{a}$$

For the commutators between generators that have a state in common, i.e., 1,2 and 1,3, one gets

(29) $$\left[X_{a},Y_{a}\right]=Z_{b};\;\left[X_{a},Y_{b}\right]=Z_{a};\;\left[X_{a},Y_{c}\right]=-X_{b}\;\left[X_{b},Y_{a}\right]={-Z}_{a};\;\left[X_{b},Y_{b}\right]=-Z_{b};\;\left[X_{b},Y_{c}\right]=-X_{a}\;\left[X_{c},Y_{a}\right]=-Y_{b};\;\left[X_{c},Y_{b}\right]=X_{a};\;\left[X_{c},Y_{c}\right]=0$$

If the two $SU\left(2\right)$ algebras do not have a state in common, their generators commute. This will happen for a system with N≥4. We generalize the commutation relations for the complete set of operators of an *N*-level system, $\left\{X_{k}\right\}$, as

(30) $$\left[X_{m},X_{n}\right]=c_{m,n}X_{\left[m,n\right]}$$

where *m* and *n* label a pair of generators and $c_{m,n}$ is the structure constant for the generator $X_{\left[m,n\right]}$ that results from the commutation relation. The functional form wrt, the $\left\{g_{k}\left(t\right)\right\}$ of the time correlation matrix Ξ defined in Equation (9), as well as of the time-correlation matrix that propagates the dynamical symmetries, Equation (12), only depend on the commutation relations Equation (30). They are valid for any Hamiltonian of the form of Equation (3).

The building blocks for building these time correlation matrices are the factors $\exp\left(g_{m}adX_{m}\right)X_{n}.$ Expanding $\exp\left(g_{m}adX_{m}\right)X_{n}$ in a Taylor series and using commutator relations, such as Equations (28) and (29), one gets the following expression

(31) $$\begin{array}{r} \exp\left(g_{m}adX_{m}\right)X_{n}=\\ X_{n}+\frac{c_{m,n}}{c_{m,\left[m,n\right]}}sin\left(c_{m,\left[m,n\right]}g_{m}\right)X_{\left[m,n\right]}+\frac{c_{m,n}}{c_{m,\left[m,n\right]}}\left(1-cos\left(c_{m,\left[m,n\right]}g_{m}\right)\right)X_{\left[m,\left[m,n\right]\right]} \end{array}$$

The actual values of the coefficients $\left\{g_{k}\left(t\right)\right\}$ as a function of time are given by Equation (10), in matrix form $\dot{g}=-i\Xi^{-1}h\left(t\right)$, and do depend on the coefficients $h\left(t\right)$ of the generators of the Hamiltonian

(32) $$H\left(t\right)=\sum_{k=1}^{\nu }h_{k}\left(t\right)X_{k}$$

In Section 6 below, we present examples of *N* = 3- and 9-level systems, where the levels are coupled through the dipole interaction with the time-dependent electric field of a light pulse, $-E\left(t\right).\;\mu$. In the Gelfand basis of generators, $E_{nm}$, the Hamiltonian takes the form

$$H\left(t\right)=\sum_{n=1,m=1}^{N}H_{nm}\left(t\right)E_{nm}$$

With

$$H_{mn}\left(t\right)=\left\{\begin{array}{ll} \alpha_{n} & \mathrm{for}\;n=m\\ -E\left(t\right)\mu_{nm} & \mathrm{for}\;n\neq m \end{array}\right.$$

The diagonal coefficients $H_{nn}(t)$ corresponds to the energies of the levels, with the lowest energy, $\alpha_{1}$ = 0 so that $\alpha_{n}$, *n* > 1, are the transition energies from the ground state to the excited states. The coefficients, $h_{k}(t)$ of the generators in Equation (32) are obtained from the following relations:

(33) $$h_{k}\left(t\right)=\left\{\begin{array}{ll} iE\left(t\right)\mu_{nm} & \mathrm{for}\;\mathrm{Mod}\left(k-1,\;3\right)=0\;\\ 0 & \mathrm{for}\;\mathrm{Mod}\left(k-2,\;3\right)=0\;\\ i\frac{1}{N}\left(-\alpha_{n}+\alpha_{m}\right) & \mathrm{for}\;\mathrm{Mod}\left(k,\;3\right)=0\; \end{array}\right.$$

Once the values of the $\left\{g_{k}\left(t\right)\right\}$ have been computed by integrating Equation (10), we can readily compute the coefficients of the time-correlation matrix ***A***(t) **=** ${\{a}_{km}(t)\}$, Equation (12), from the Schrödinger picture to the dynamical symmetries:

(34) $$X_{k}(t)=UX_{k}U^{-1}=\left(\prod_{j=1}^{\nu }\exp\left(g_{j}\left(t\right)adX_{j}\right)\right)X_{k}=\sum_{m=1}^{\nu }a_{km}(t)X_{m}$$

From ***A***(*t*), one also readily obtains the coefficients of the time correlation matrix from the Schrödinger picture to the Heisenberg operators, ***B****(t)* **=** ${\{b}_{km(t)}\}$, which is the inverse of ***A***(*t*) or compute them directly using:

(35) $$X_{k}\left(t\right)=X_{k}(-t)=U^{-1}X_{k}U=\left(\prod_{j=\nu }^{1}\exp\left(-g_{j}\left(t\right)adX_{j}\right)\right)X_{k}=\sum_{m=1}^{\nu }b_{km}{\left(t\right)X}_{m}$$

See Supplementary Section S2 for more details.

## 6. Applications to the Electronic Dynamics of CdSe Nanoparticles

### 6.1. Electronic Dynamics for a Single CdSe Nanoparticle: An N = 3 Model

**Figure 1:**
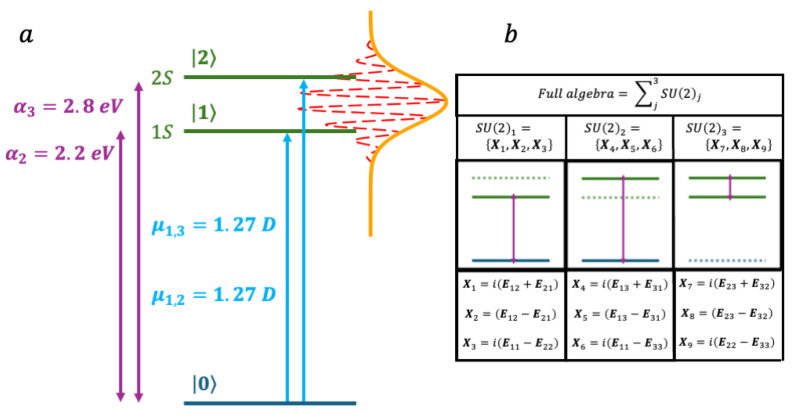
(**a**) Level structure of the three-state model of a CdSE nanoparticle. Two excited electronic states, 1S and 2S are optically coupled to the ground state. (**b**) The corresponding three coupled SU(2) algebras leading to 8 generators $X_{k}$.

Small CdSe nanoparticles have been studied extensively for exploiting their optical properties [64,65,66,67,68,69,70,71], and coherences between electronic states have been measured using 2 dimensional electronic spectroscopy (2DES) [54,55,72,73,74,75,76,77,78]. To illustrate our approach, we begin with a simple three-level model describing the electronic dynamics of a small 3 nm in diameter CdSe nanoparticle, optically excited by a VIS few fs laser pulse. The three electronic states that we consider are the ground electronic state (GS), and the two lowest excitonic states, 1S and 2S of the nanoparticle [54,55,65,66,79,80], made of two hole-electron pairs: the h_1_e exciton for S1 and the h_2_e exciton for 2S. 1S and 2S are 2.2 eV and 2.8 eV above the GS respectively and their transition dipole from the GS are set equal: ${\;\mu }_{12}=\mu_{13}$ = 1.27 Debye (0.5 a.u.). The level structure is shown in Figure 1.

This model is relevant when the spectroscopic energy resolution used to probe the electronic dynamics is not high enough to resolve the fine structure of excited states 1S and 2S.

The CdSe nanoparticle is addressed by a sequence of three identical fs pulse with a Gaussian envelope in time broad enough in time to have a sufficient large energy bandwidth for exciting the states 1S and 2S and building electronic coherences, as is done in the 2DES [54,55,72,73,74,75,76,77,78] for probing electronic coherences in view of application to quantum technologies [43]:

(36) $$E\left(t\right)=E_{0}\exp\left(\frac{{-\left(t-t_{0}\right)}^{2}}{2\sigma^{2}}\right)\cos\left(\omega_{c}(t-t_{0}\right))$$

with a Full Width at Half Maximum (FWHM) = $2\sqrt{2ln2}\sigma =$ 5.86 fs, a carrier frequency, $\omega_{c}$ of 2.5 eV. The strength of the electric field, $E_{0}$ is set to 0.007 a.u.

This three-level system corresponds to three coupled qubits, described by three coupled $SU\left(2\right)$ algebras with nine generators, three for each pair of levels: $\left\{X_{1},X_{2},X_{3}\right\}$ for the GS and 1S, $\left\{X_{4},X_{5},X_{6}\right\}$ for the GS and 2S and $\left\{X_{7},X_{8},X_{9}\right\}$ for 1S and 2S. Note that, in this case, the three pairs of levels each have a state in common. The commutation relations between the nine operators are given in Table S3 of the Supplementary as well as the explicit functional form of the three-state time correlation matrix B (Equation (35), see Supplementary, Section S3.3)) and an example of the dynamical symmetries’ dependence on of the $\left\{g_{k}\left(t\right)\right\}$ (Equation (34), see Supplementary Section S3.4).

In term of the $\{X_{k}$}, the Hamiltonian takes the form, see Equation (33)

$$H\left(t\right)=\frac{1}{3}\left(\alpha_{2}+\alpha_{3}\right)I+iE\left(t\right)\mu_{12}X_{1}+i\frac{\alpha_{2}}{3}X_{3}+iE\left(t\right)\mu_{13}X_{4}+i\frac{\alpha_{3}}{3}X_{6}+\frac{1}{3}i\left(-\alpha_{2}+\alpha_{3}\right)X_{9}$$

**Figure 2:**
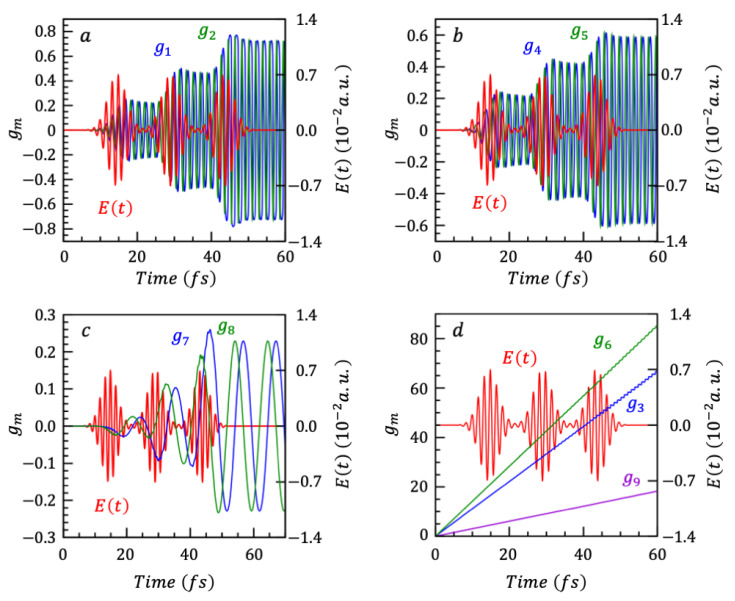
The nine $g_{i}$ which govern the evolution operator U (Equation (6)) of the three-state system. Panels (**a**–**c**) show $g_{i}$ which are associated with coherence operators, and panel (**d**) shows $g_{i}$ which are associated with the population difference operators. All panels also show the three pulses $E\left(t\right)$ applied at $t_{0}=14.5\;fs,$ 29 fs and 43.5 fs.

We show in Figure 2a–c the computed time dependence of the pairs of g coefficients, $\left\{g_{1}\left(t\right),g_{2}\left(t\right)\right\}$, $\left\{g_{4}\left(t\right),g_{5}\left(t\right)\right\}$ and $\left\{g_{7}\left(t\right),g_{8}\left(t\right)\right\}$ that correspond to the pairs of generators $\left\{X_{1},X_{2}\right\}$, $\left\{X_{4},X_{5}\right\}$ and $\left\{X_{7},X_{8}\right\}$ describing the real and imaginary parts of the electronic coherences. $g_{3}\left(t\right),\;g_{6}\left(t\right)$ and $g_{9}\left(t\right)$ which corresponds to the operators associated with the population differences $X_{3}$**,** $X_{6}$ and $X_{9}$ are plotted in Figure 2d. After the pulse, the $\left\{g_{1}\left(t\right),g_{2}\left(t\right)\right\}$, $\left\{g_{4}\left(t\right),g_{5}\left(t\right)\right\}$ and $\left\{g_{7}\left(t\right),g_{8}\left(t\right)\right\}$ coefficients oscillate between constant values while the $g_{3}\left(t\right),\;g_{6}\left(t\right)$ and $g_{9}\left(t\right)$ increase monotonically with a step-fine structure.

The oscillations that appear in each $\left\{g_{n}\left(t\right),g_{m}\left(t\right)\right\}$ are determined by the energies of states coupled by the SU(2) to which this pair corresponds. When an SU(2) couples two states i and j, the $\left\{g_{n}\left(t\right),g_{m}\left(t\right)\right\}$ of this SU(2) will contain oscillations related to the frequency $∆\alpha_{i,j}$ and to the $∆\alpha_{k,j}$ of all states k between i and j.

The values of $\left\{g_{k}\left(t\right)\right\}$ coefficients only depend on the Hamiltonian. Once the $\left\{g_{k}\left(t\right)\right\}$ have been determined one can readily compute the constant values of the dynamical symmetries, $\left\langle X_{k}(t\right)\rangle =Tr\;[\rho \left(t\right)\;X_{k}\left(t\right)]$ and the time-dependent values of the Schrödinger operators $\left\langle X_{k}(t)\right\rangle =Tr\;[\rho \left(t\right)\;X_{k}]$ for a given initial state. Both can be computed by determining the time correlation matrices from the Schrödinger picture to the dynamical symmetries, Equation (34), and to the Heisenberg operators, Equation (35) in terms of the $\left\{g_{k}\left(t\right)\right\}.$ Since we describe the response of the nanoparticle to an optical excitation, the initial state is the ground electronic state. The latter has coefficients on two generators only, $X_{3}$ and $X_{6}$, with equal initial mean values $\left\langle X_{3}(0)\right\rangle =i$ and $\left\langle X_{6}(0)\right\rangle =i$. All other initial mean values are 0. It means that for this particular initial state, two dynamical symmetries only, $X_{3}(t)$ and $X_{6}(t)$, have a constant mean value different from 0: $\left\langle X_{3}(0)\right\rangle =\left\langle X_{6}(0)\right\rangle =i$, all the other $\langle X_{k}(0)\rangle =0$ for k≠3, 6. For such an initial state, which has coefficients on a limited number of generators, there is a considerable saving of computer time and storage for propagating the mean values $\langle X_{k}\left(t\right)\rangle$ of the generators corresponding to the coherences and to the population differences. This can be seen explicitly from Equation (37) below which gives the time evolution of the mean values of the nine generators $\left\{\langle X_{k}\left(t\right)\rangle \right\}$ using Equation (35):

(37) $$\left(\begin{array}{c} \langle X_{1}\left(t\right)\rangle \\ \langle X_{2}\left(t\right)\rangle \\ \langle X_{3}\left(t\right)\rangle \\ \langle X_{4}\left(t\right)\rangle \\ \langle X_{5}\left(t\right)\rangle \\ \langle X_{6}\left(t\right)\rangle \\ \langle X_{7}\left(t\right)\rangle \\ \langle X_{8}\left(t\right)\rangle \\ \langle X_{9}\left(t\right)\rangle \end{array}\right)=\left(\begin{array}{ccccccccc} b_{11} & b_{12} & b_{13} & b_{14} & b_{15} & b_{16} & b_{17} & b_{18} & b_{19}\\ b_{21} & b_{22} & b_{23} & b_{24} & b_{25} & b_{26} & b_{27} & b_{28} & b_{29}\\ b_{31} & b_{32} & b_{33} & b_{34} & b_{35} & b_{36} & b_{37} & b_{38} & b_{39}\\ b_{41} & b_{42} & b_{43} & b_{44} & b_{54} & b_{46} & b_{47} & b_{48} & b_{49}\\ b_{51} & b_{52} & b_{53} & b_{54} & b_{55} & b_{56} & b_{57} & b_{58} & b_{59}\\ b_{61} & b_{62} & b_{63} & b_{64} & b_{65} & b_{66} & b_{67} & b_{68} & b_{69}\\ b_{71} & b_{72} & b_{73} & b_{74} & b_{75} & b_{76} & b_{77} & b_{78} & b_{79}\\ b_{81} & b_{82} & b_{83} & b_{84} & b_{85} & b_{86} & b_{87} & b_{88} & b_{89}\\ b_{91} & b_{92} & b_{93} & b_{94} & b_{95} & b_{96} & b_{97} & b_{98} & b_{99} \end{array}\right)\cdot \left(\begin{array}{c} 0\\ 0\\ i\\ 0\\ 0\\ i\\ 0\\ 0\\ 0 \end{array}\right)$$

**Figure 3:**
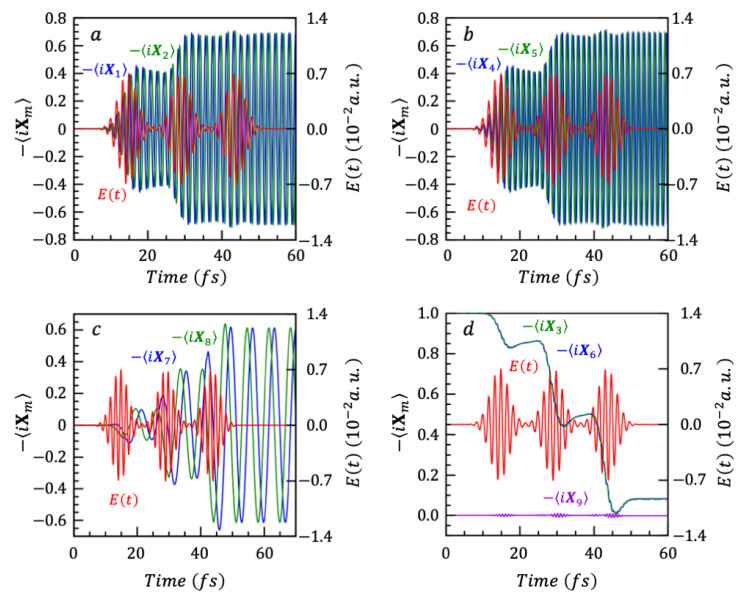
The nine $\left\langle X_{k}\left(t\right)\right\rangle$ of the three-state system, plotted as $-\left\langle iX_{k}(t)\right\rangle$. Panels (**a**–**c**) show $-\left\langle iX_{k}\right\rangle (t)$ of coherence operators, and panel (**d**) shows the $-\left\langle iX_{k}(t)\right\rangle$ of the population difference operators. All panels also show the sequence of three pulses $E\left(t\right)$, which drives the dynamics.

The time evolution of the mean values of the nine observables is given by two columns of the time correlation matrix only, the 3rd and the 6th, marked in gray shade in Equation (37), meaning only 18 coefficients need to be derived. Their derivation is further simplified by exploiting the commutation relations between the $\left\{X_{k}\right\}$ generators, as explained in Section S3.3 of the Supplementary where their explicit expressions in terms of the $\left\{g_{k}\left(t\right)\right\}$ coefficients are given. The resulting real and imaginary parts of the coherences and the population differences are plotted in Figure 3 as a function of time. Note that it is the excitation by the second pulse that builds coherence between states 2 and 3. The population difference $X_{9}(t)$ shown in Figure 3d, between states 1S and 2S is small because the two states are about equally populated. This can be seen from the amplitude of the oscillations of the real and imaginary parts of coherence between these two states shown in Figure 3c.

### 6.2. Electronic Dynamics in a Nine-Level Dimer of CdSe Nanoparticles

**Figure 4:**
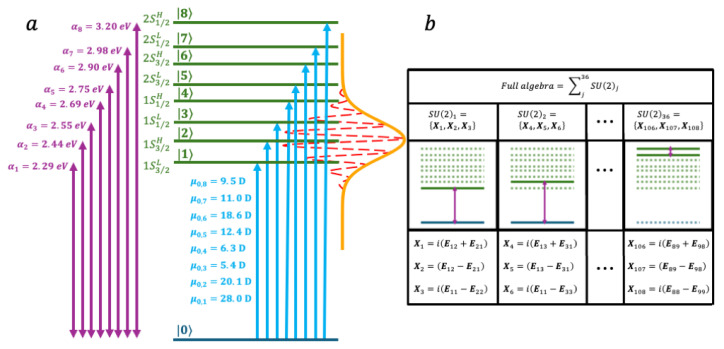
(**a**) Level structure of the dimer of Cdse nanoparticles. The ground states are coupled to eight excited states ($1S_{3/2}^{L}$, $1S_{3/2}^{H}$, $1S_{1/2}^{L}$, $1S_{1/2}^{H}$, $2S_{3/2}^{L}$, $2S_{3/2}^{H}$, $2S_{1/2}^{L}$ and $2S_{1/2}^{H}$) by optical excitation. (**b**) the generators $\left\{X_{k}\right\}$ of the nine-level system is constituted of 36 SU(2) algebras.

**Figure 5:**
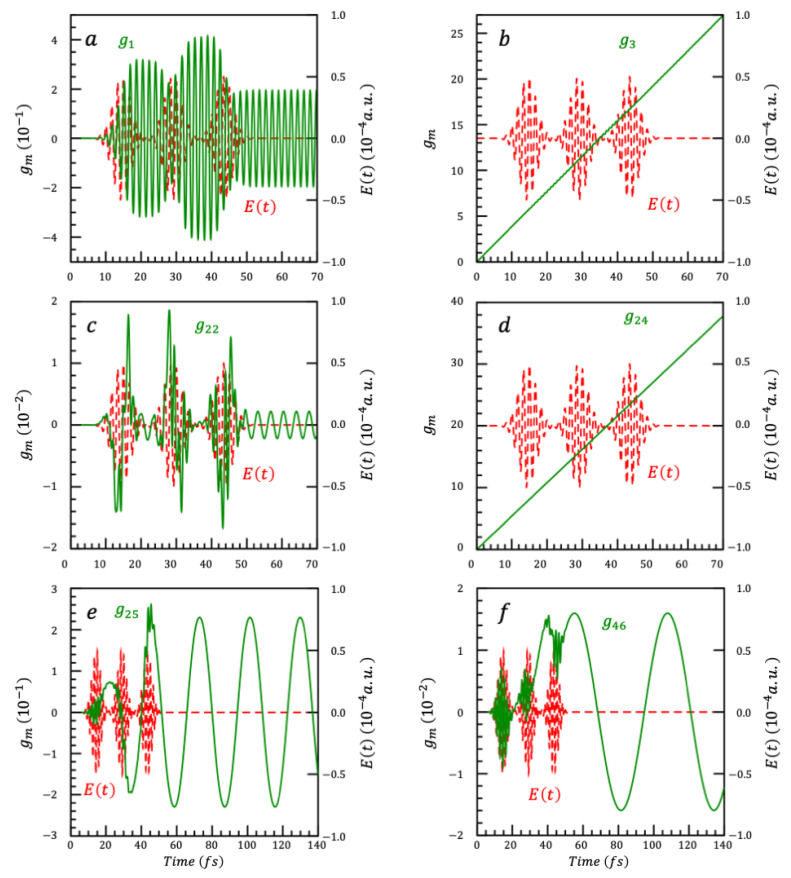
Six of the 108 $g_{i}$ which govern the time evolution operator U (Equation (6)) of the 9-state system. Panels (**a**,**c**,**e**,**f**) show $g_{i}$ which are associated with coherence generators, and panels (**b**,**d**) show $g_{i}$ which are associated with population difference generators. Panel (**a**) shows $g_{1}$, which is associated with $X_{1}=i\left(E_{12}+E_{21}\right)$; Panel (**b**) shows $g_{3}$, which is associated $X_{3}=i\left(E_{11}-E_{22}\right)$; Panel (**c**) shows $g_{22}$, which is associated with $X_{22}=i\left(E_{18}+E_{81}\right)$; Panel (**d**) shows $g_{24}$, which is associated with $X_{24}=i\left(E_{11}-E_{88}\right)$; panel (**e**) shows $g_{25}$, which is associated with $X_{25}=i\left(E_{23}+E_{32}\right)$; and panel (**f**) shows $g_{46}$, which is associated with $X_{46}=i\left(E_{34}+E_{43}\right).$ All panels also show the three pulses $E\left(t\right)$.

We next consider a realistic model of a dimer of small, ≈ 3 nm in diameter, CdSe nanoparticles [55,81]. In this model, we take into account the spin-orbit coupling between the spin of the hole and its orbital quantum number in computing the energies of the excitons for each CdSe nanoparticle [54,65,66] Each dimer has therefore 8 excited states, in the energetic order $1S_{3/2}^{L}$, $1S_{3/2}^{H}$, $1S_{1/2}^{L}$, $1S_{1/2}^{H}$, $2S_{3/2}^{L}$, $2S_{3/2}^{H}$, $2S_{1/2}^{L}$ and $2S_{1/2}^{H}$. In refs [55,81], we show that the electronic coherences between these excited states can be probed by coherent 2DES. The ladder of 9 states (8 excited states + the ground state) is shown in Figure 4, as well as the structure of the corresponding 36 coupled SU(2) algebra, leading to 108 generators $\left\{X_{k}\right\}$ and 108 $g_{k}(t)$. We here apply a sequence of 3 fs laser pulses (Equation (36)) at 14.5 fs, 29 fs, and 43.5 fs. The three pulses have the same parameters: FWHM = $2\sqrt{2ln2}\sigma =$ 5.86 fs, a carrier frequency, $\omega_{c}$ of 2.45 eV, and the electric field strength, $E_{0}$, is set to 0.003 a.u. Figure 5 shows the time evolution of the selected values of the $\left\{g_{k}\left(t\right)\right\}$.

**Figure 6:**
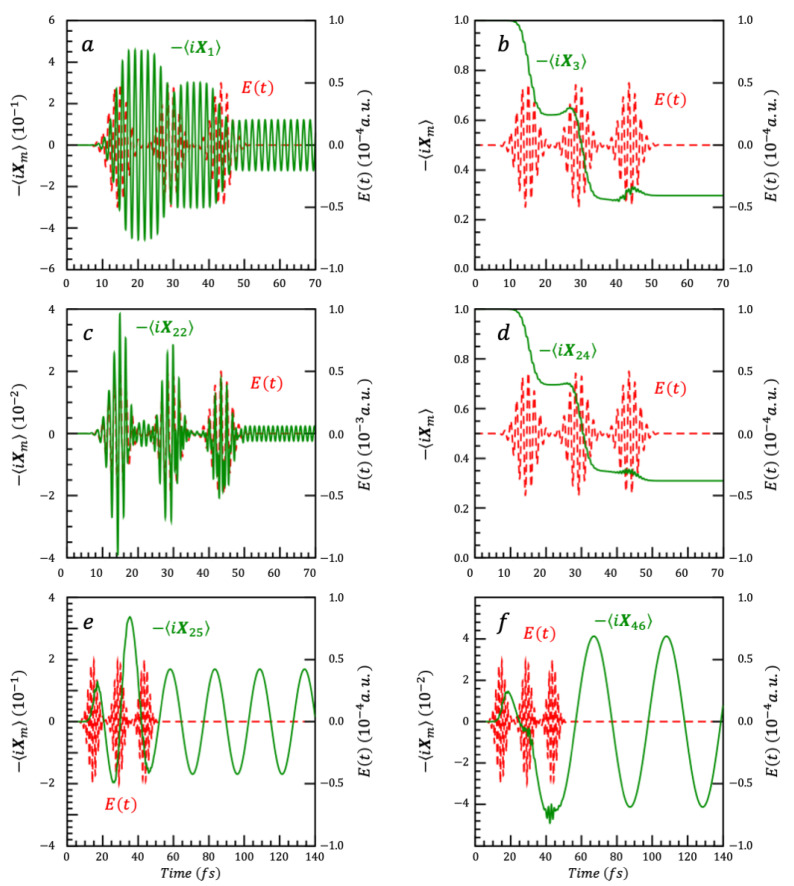
Six of the 108 $\left\langle X_{k}(t)\right\rangle$ of the nine-state system, plotted as $-\left\langle iX_{k}(t)\right\rangle$. Panels (**a**,**c**,**e**,**f**) show $-\left\langle iX_{k}(t)\right\rangle$ of coherence operators, and panels (**b**,**d**) show $-\left\langle iX_{k}(t)\right\rangle$ of population difference operators. All panels also show the time profile of the electric field, $E\left(t\right)$, made of a sequence of three pulses, which drives the dynamics.

Since the initial state is the ground state, only two dynamical symmetries have a mean value different from 0, $\left\langle X_{3}(0)\right\rangle =\left\langle X_{6}(0)\right\rangle =i$, all the other $\left\langle X_{k}(0)\right\rangle ’s$ = 0. As for the three-level system discussed in Section 6.1, only two columns, the third and the sixth ones, of the time correlation from the Schrödinger to the Heisenberg picture, Equation (35), need to be derived to propagate the observables, meaning 216 coefficients out of 108^2^ = 11,664. The time dependence of $\left\langle X_{k}(t)\right\rangle$ corresponding to the $g_{k}\left(t\right)$ plotted in Figure 5 are plotted in Figure 6. Note that each $\left\langle X_{k}(t)\right\rangle$ depends on several $g_{k}\left(t\right)$.

## 7. Conclusions and Perspectives

A practical approach to computing quantum dynamical symmetries is discussed and implemented for systems of coupled two states (=qubits), which provides a direct connection to quantum computing designs implemented on currently available quantum hardware [82] and to Ising Hamiltonians [83,84]. To address the optical, the Hamiltonian can be written as ($H=\sum_{i,j}H_{ij}\left|i\right\rangle \left\langle j\right|\;$), and it is of an Ising form when each quantum level is encoded on the spin state of a qubit. One can choose the three generators for each *SU*(2) algebra such that the factorized evolution operator is guaranteed unitary. The factorization is successive. Each *SU*(2) algebra results in its own factor in the evolution operator, and each such factor is a sequence of terms, one for each of the three generators (see Table 1). As reported in detail in the Supplementary Materials and shown in the examples discussed in Section 6, this leads to a very stable and compact numerical scheme, even for strong coupling, and to considerable savings in terms of computer time and storage.

Beyond the application to computing with coupled qubits, the methodology we discussed can be applied to many aspects of dynamical systems. This is because we compute the evolution operator, which is valid for any initial state. The one restriction is that in this paper, we compute a unitary evolution operator so that we do not describe dissipation. From the evolution operator, we generate the dynamical symmetries, Section 2. The key point towards applications is that a function of dynamical symmetries is itself a dynamical symmetry.

As a practical application, we compute how expectation values that are needed to describe the system can be computed without appeal to a wave function. This is the role of the time correlation matrix that relates initial values to the expectation values of observables at time *t*. Its expectation value at time *t* is explicitly given as an expectation value of a dynamical symmetry over *the initial state.*

$\langle A_{r}(t)\rangle \equiv Tr\left(\rho \left(0\right)U^{†}\left(t\right)A_{r}\left(0\right)U(t)\right)=Tr\left(\rho \left(0\right)A_{r}\left(-t\right)\right)=Tr\left(\rho \left(0\right)\sum_{s}A_{s}a_{rs}\left(-t\right)\right)=\sum_{s}\langle A_{s}\rangle \left(t=0\right)a_{rs}\left(-t\right)$. It is most practical to determine dynamical symmetries when the Hamiltonian is specified as a matrix, as we do in this paper. In this case, the states of the system are pairwise coupled and the dynamics can be cast as coupled two-state systems.

Dynamical symmetries are used not only to describe the dynamics of operators. They also play a key role in the complementary task of propagating the *state* of the system in time. This is because the density operator under a unitary time evolution is itself a dynamical symmetry. A simple example is when the initial state is, say, the ground state, $\left.A_{g}≝\rho \left(0\right)=|i\right\rangle \left\langle i\right.|\;$ then the state at a later time is $\rho \left(t\right)=A_{g}\left(t\right)$. In a more general case, when the initial state is specified by a number of operators $\rho \left(0\right)=f(\left\{A_{i}\right\})$, then the unitarity of the evolution operator implies that at a later time $\rho \left(t\right)=f(\left\{A_{i}\left(-t\right)\right\})$. The operators $\left\{A_{i}\right\}$ can be the set of skew Hermitian opertators $\left\{X_{i}\right\}$ of Section 6 or any Lie closed set. An important special case is when the initial state is a density operator of maximal entropy. Then the set $\left\{A_{i}\right\}$ is the set operator whose mean value is given and it constraints the entropy. It follows that at all subsequent times, the density matrix is of maximal entropy subject to the mean values of the dynamical symmetries, see [28,30]. Of course, the mean values of the dynamical symmetries remain unchanged in time. But Equation (4), $A_{r}(t)=\sum_{s}a_{rs}(t)A_{s}$ shows how the state at time *t* can be expressed in terms of the time independent Schrödinger picture operators $\rho \left(t\right)=f(\left\{\sum_{s}a_{rs}(-t)A_{s}\right\}).$ Explicit results for systems with coherences show that the distribution of populations at time *t* is not necessarily simple exponentials and is often a sum of such terms. It may be of interest to draw an analogy with classical distributions of maximal entropy. These can also be not a single exponential, for example, when there are several paths leading to the same final state. If $P\left(j,n\right)$ is the probability of state *j* via the distinct path *n* and $P\left(j\right)$ is the total probability of the state *j*, then $P\left(j\right)=\sum_{n}P\left(j,n\right)$. It remains to be clearly understood whether this is analogous to the inherently parallel processing in quantum computing.

## Figures and Tables

**Table 1:** Number, η, of directly coupled SU(2) algebras for systems of N quantum states and number of generators, ν.

N	$\eta =N\left(N-1\right)/2$	ν=3η
N=2	η=1	ν=3
N=3	η=3	ν=9
N=4	η=6	ν=18

## Data Availability

Data are contained within the article and Supplementary Materials.

## Supplementary Materials

The following supporting information can be downloaded at: https://www.mdpi.com/article/10.3390/nano14242056/s1, The Supplementary Material provides the details of the derivations of the results of the coupled two-state *SU*(2) system discussed in the main text and for the three- and nine-coupled states models.
