# Supplementary material for: Constructing Dynamical Symmetries for Quantum Computing: Applications to Coherent Dynamics in Coupled Quantum Dots
Source: Nanomaterials (Basel). 2024 Dec 23;14(24):2056. doi: 10.3390/nano14242056 (PMC11677228; doi:10.3390/nano14242056)
Supplement: Supplementary file 1 [file nanomaterials-14-02056-s001.zip › nanomaterials-3336618-supplementary.pdf]

Constructing Dynamical Symmetries for quantum computing: Applications to coherent dynamics in coupled quantum dots

J. R. Hamilton[1,2], R. D. Levine[2,3,4] , F. Remacle[1,2,*]
[1]Theoretical Physical Chemistry, UR MOLSYS, University of Liege, Belgium
[2]Institute of Chemistry, The Hebrew University of Jerusalem, Jerusalem 91904, Israel
[3] Department of Molecular and Medical Pharmacology, David Geffen School of Medicine
and
[4] Department of Chemistry and Biochemistry, University of California, Los Angeles, CA 90095, USA

Supplementary Material

Table of content

S1. Additional details on the two state system
S2. The N-State Generalisation
S3. CdSe nanoparticles models
S4. References

S1. Additional details on the two state system

In this section we provide additional details on the solution of the two state problem discussed in section 3 of the main text.

The Wei- Norman parametrization of the matrix time evolution operator of the two-state system is given by equation (6) of the main text: [1,2]

$$\boldsymbol{U} = \exp(g_1(t)\boldsymbol{X}_1)\exp(g_2(t)\boldsymbol{X}_2)\exp(g_3(t)\boldsymbol{X}_3) \qquad (S1)$$

Our experience is that a more practical approach calls for the set of operators $\{\boldsymbol{X}_i\}$ to be a skew-Hermitian basis as was proposed by Altafini [3,4] and used in ref. [5] up to a factor ½, see Eq. (13) to (16) of the main text.

The Hamiltonian for two state system subject to an optical excitation by a laser pulse *E*(t) is given by equations (17) to (20) of the main text, where $\boldsymbol{X}_3$ is the population difference

$\boldsymbol{H} = iE(t)\mu\boldsymbol{X}_1 + i\frac{\alpha}{2}\boldsymbol{X}_3 + \frac{\alpha}{2}\boldsymbol{I}$

So that the vector of the 3 coefficients in equation (3) of the main text, $\boldsymbol{H} = \sum_r h_r(t)\boldsymbol{X}_r$, are:

$$\boldsymbol{h} \equiv \begin{pmatrix} h_1 \\ h_2 \\ h_3 \end{pmatrix} = \begin{pmatrix} iE(t)\mu \\ 0 \\ i\,\alpha/2 \end{pmatrix} \quad \text{(S2)}$$

S1.1 Calculating the two state $\{\boldsymbol{g}_k(t)\}$

This section summarizes how to construct the correlation matrix $\Xi(t)$ and obtain the equation of motions for the time-dependent $\{g_k(t)\}$ parameters of the evolution operator (equation (S1)) following the approach of Wei and Norman. [1,2]

As derived in Section 3 of the main text, the equation that needs to be solved to get the time dependence of the $\{g_k\}$ coefficients in Eq. (S1) is the non linear equation

$$\dot{\boldsymbol{g}} = -i\,\Xi^{-1}(\boldsymbol{g})\cdot\mathbf{h} \quad (S3)$$

The columns of Ξ are therefore calculated from the $\{\boldsymbol{X}_k\}$ with commutator algebra. Table S1 gives the commutation relations of the two state $\{\boldsymbol{X}_k\}$ basis.

Table S1: The commutation relations for the two state $\{\boldsymbol{X}_k\}$ basis

	$[\cdot,\boldsymbol{X}_1]$	$[\cdot,\boldsymbol{X}_2]$	$[\cdot,\boldsymbol{X}_3]$
$[\boldsymbol{X}_1,\cdot]$	0	$-2\boldsymbol{X}_3$	$2\boldsymbol{X}_2$
$[\boldsymbol{X}_2,\cdot]$	$2\boldsymbol{X}_3$	0	$-2\boldsymbol{X}_1$
$[\boldsymbol{X}_3,\cdot]$	$-2\boldsymbol{X}_2$	$2\boldsymbol{X}_1$	0

Expanding $\exp(g_n ad\mathbf{X}_n)\,\mathbf{X}_m$ in a Taylor series

$$\exp(g_n ad\boldsymbol{X}_n)\boldsymbol{X}_m = \boldsymbol{X}_m + g_n[\boldsymbol{X}_n,\boldsymbol{X}_m] + \frac{(g_n)^2}{2!}[\boldsymbol{X}_n,[\boldsymbol{X}_n,\boldsymbol{X}_m]] + \frac{(g_n)^3}{3!}\big[\boldsymbol{X}_n,[\boldsymbol{X}_n,[\boldsymbol{X}_n,\boldsymbol{X}_m]]\big]\ldots$$

(S4)

and using the commutator relations from table $(S1)$, one obtains the following $\exp(g_n ad\boldsymbol{X}_n)\,\boldsymbol{X}_m$ are needed to construct the Ξ matrix:

$$\exp(g_2 ad\boldsymbol{X}_2)\boldsymbol{X}_3 = \cos(2g_2)\,\boldsymbol{X}_3 - \sin(2g_2)\,\boldsymbol{X}_1 \quad (S5)$$

$$\exp(g_1 ad\boldsymbol{X}_1)\boldsymbol{X}_3 = \cos(2g_1)\,\boldsymbol{X}_3 + \sin(2g_1)\,\boldsymbol{X}_2 \quad (S6)$$

$$\exp(g_1 ad\boldsymbol{X}_1)\boldsymbol{X}_2 = \cos(2g_1)\,\boldsymbol{X}_2 - \sin(2g_1)\,\boldsymbol{X}_3 \quad (S7)$$

$$\exp(g_1 ad\boldsymbol{X}_1)\boldsymbol{X}_1 = \boldsymbol{X}_1 \tag{S8}$$

Therefore, the elements of the $\Xi(t)$ matrix are calculated a-la Wei-Norman as explained in ref. [5]and in Altafini[3,4]. We reproduced here the different steps for completeness.

By definition, the elements of the 1st column of Ξ are

$\xi_{11} = 1$

and

$\xi_{1m} = 0\ \forall$ other m.

Using equations ($S6$) and ($S7$), the column 2 of Ξ matrix is given by $\exp(g_1 ad\boldsymbol{X}_1)\boldsymbol{X}_2$, so from equation ($S4$), $\exp(g_1 ad\boldsymbol{X}_1)\boldsymbol{X}_2 = \cos(2g_1)\,\boldsymbol{X}_2 - \sin(2g_1)\,\boldsymbol{X}_3$, one obtains the elements of the 2nd column

$\xi_{22} = \cos(2g_1)$

and

$\xi_{32} = -\sin(2g_1)$

Using equations ($S5$) – ($S8$), column 3 of the Ξ matrix is given by

$\exp(g_2 ad\boldsymbol{X}_2)\boldsymbol{X}_3 = \cos(2g_2)\,\boldsymbol{X}_3 - \sin(2g_2)\,\boldsymbol{X}_1$

$\exp(g_1 ad\boldsymbol{X}_1)\exp(g_2 ad\boldsymbol{X}_2)\boldsymbol{X}_3 = \cos(2g_2)\exp(g_1 ad\boldsymbol{X}_1)\boldsymbol{X}_3 - \sin(2g_2)\exp(g_1 ad\boldsymbol{X}_1)\boldsymbol{X}_1$

$\exp(g_1 ad\boldsymbol{X}_1)\exp(g_2 ad\boldsymbol{X}_2)\boldsymbol{X}_3 = -\sin(2g_2)\,\boldsymbol{X}_1 + \cos(2g_2)\sin(2g_1)\,\boldsymbol{X}_2 +$
$\cos(2g_2)\cos(2g_1)\,\boldsymbol{X}_3$

Therefore, the elements of the 3rd column are

$\xi_{13} = -\sin(2g_2)$

$\xi_{23} = \cos(2g_2)\sin(2g_1)$

and

$\xi_{33} = \cos(2g_2)\cos(2g_1)$

Arranging the columns into a matrix, one get the $\Xi(t)$ matrix

$$\Xi = \begin{pmatrix} 1 & 0 & -\sin(2g_2) \\ 0 & \cos(2g_1) & \cos(2g_2)\sin(2g_1) \\ 0 & -\sin(2g_1) & \cos(2g_2)\cos(2g_1) \end{pmatrix} \tag{S9}$$

For the two state system, the inverse of Ξ can be obtained analytically:

$$\boldsymbol{\Xi^{-1}} = \begin{pmatrix} 1 & \tan(2g_2)\sin(2g_1) & \tan(2g_2)\cos(2g_1) \\ 0 & \cos(2g_1) & -\sin(2g_1) \\ 0 & \sec(2g_2)\sin(2g_1) & \sec(2g_2)\cos(2g_1) \end{pmatrix} \tag{S10}$$

Explicitly using equation ($S10$) for $\boldsymbol{\Xi^{-1}}$ in equation ($S3$) we get

$$\begin{pmatrix} \dot{g}_1 \\ \dot{g}_2 \\ \dot{g}_3 \end{pmatrix} = \begin{pmatrix} 1 & \tan(2g_2)\sin(2g_1) & \tan(2g_2)\cos(2g_1) \\ 0 & \cos(2g_1) & -\sin(2g_1) \\ 0 & \sec(2g_2)\sin(2g_1) & \sec(2g_2)\cos(2g_1) \end{pmatrix} \begin{pmatrix} E(t)\mu \\ 0 \\ \alpha/2 \end{pmatrix}$$

Which is a matrix representation of a system of coupled equations which can be solved numerically to get the $\{g_k\}$. In this work, the numerical integration is done using the Cash-Karp Runge-Kutta method [7].

1.2 Calculating the two state time evolution operator

Having the equations to calculate the $\{g_k\}$, one needs a matrix representation of the time evolution operator $\boldsymbol{U}$ which can be used to calculate the time evolution of the observables.

We expand each factor of $\mathbf{U}$ in equation $(S1)$, $\boldsymbol{U} = \exp(g_1\boldsymbol{X}_1)\exp(g_2\boldsymbol{X}_2)\exp(g_3\boldsymbol{X}_3)$ as a Taylor series. Starting with $\exp(g_1\boldsymbol{X}_1)$:

$$\exp(g_1\boldsymbol{X}_1) = \boldsymbol{I} + g_1\boldsymbol{X}_1 + \frac{(g_1\boldsymbol{X}_1)^2}{2!} + \frac{(g_1\boldsymbol{X}_1)^3}{3!} + \frac{(g_1\boldsymbol{X}_1)^4}{4!} + \frac{(g_1\boldsymbol{X}_1)^5}{5!} \ldots$$

with

$$(\boldsymbol{X}_1)^\beta = \begin{cases} (i)^\beta(\boldsymbol{E}_{12} + \boldsymbol{E}_{21}) & \text{for } \beta \in \{1,3,5,7\ldots\} \\ (i)^\beta(\boldsymbol{E}_{11} + \boldsymbol{E}_{22}) & \text{for } \beta \in \{2,4,6,8\ldots\} \end{cases}$$

and $\boldsymbol{I} = \boldsymbol{E}_{11} + \boldsymbol{E}_{22}$, the $\exp(g_1\boldsymbol{X}_1)$ series gives:

$$\exp(g_1\boldsymbol{X}_1) = \boldsymbol{E}_{11} + \boldsymbol{E}_{22} + g_1(i)^1(\boldsymbol{E}_{12} + \boldsymbol{E}_{21}) + \frac{(g_1)^2(i)^2(\boldsymbol{E}_{11}+\boldsymbol{E}_{22})}{2!} + \frac{(g_1)^3(i)^3(\boldsymbol{E}_{12}+\boldsymbol{E}_{21})}{3!} + \frac{(g_1)^4(i)^4(\boldsymbol{E}_{11}+\boldsymbol{E}_{22})}{4!} + \frac{(g_1)^5(i)^5(\boldsymbol{E}_{12}+\boldsymbol{E}_{21})}{5!} \ldots$$

This can be rearranged:

$$\exp(g_1\boldsymbol{X}_1) = \left(1 - \frac{(g_1)^2}{2!} + \frac{(g_1)^4}{4!} \ldots\right)(\boldsymbol{E}_{11} + \boldsymbol{E}_{22}) + i\left(g_1 - \frac{(g_1)^3}{3!} + \frac{(g_1)^5}{5!} \ldots\right)(\boldsymbol{E}_{12} + \boldsymbol{E}_{21})$$

This shows $\exp(g_1\boldsymbol{X}_1)$ can be written out as a linear combination of $\boldsymbol{E}_{ij}$ and therefore as a matrix:

$$\exp(g_1\boldsymbol{X}_1) = \left(\cos(g_1)(\boldsymbol{E}_{11} + \boldsymbol{E}_{22}) + i\sin(g_1)(\boldsymbol{E}_{12} + \boldsymbol{E}_{21})\right) = \begin{pmatrix} \cos(g_1) & i\sin(g_1) \\ i\sin(g_1) & \cos(g_1) \end{pmatrix} \quad (S11)$$

Similarly, $\exp(g_2\boldsymbol{X}_2)$ can be expanded out as a series

$$\exp(g_2\boldsymbol{X}_2) = \boldsymbol{I} + g_2\boldsymbol{X}_2 + \frac{(g_2\boldsymbol{X}_2)^2}{2!} + \frac{(g_2\boldsymbol{X}_2)^3}{3!} + \frac{(g_2\boldsymbol{X}_2)^4}{4!} + \frac{(g_2\boldsymbol{X}_2)^5}{5!} \ldots$$

Using the result for the exponent

$$(\boldsymbol{X}_2)^\beta = \begin{cases} (i)^{\beta-1}(\boldsymbol{E}_{12} - \boldsymbol{E}_{21}) & \text{for } \beta \in \{1,3,5,7\ldots\} \\ (i)^\beta(\boldsymbol{E}_{11} + \boldsymbol{E}_{22}) & \text{for } \beta \in \{2,4,6,8\ldots\} \end{cases}$$

And $\boldsymbol{I} = \boldsymbol{E}_{11} + \boldsymbol{E}_{22}$, substituting these relations into the $\exp(g_2\boldsymbol{X}_2)$ series gives:

$$\exp(g_2\boldsymbol{X}_2) = (\boldsymbol{E}_{11}+\boldsymbol{E}_{22}) + g_2(\boldsymbol{E}_{12}-\boldsymbol{E}_{21}) + \frac{(ig_2)^2(\boldsymbol{E}_{11}+\boldsymbol{E}_{22})}{2!} + \frac{(g_2)^3(i)^2(\boldsymbol{E}_{12}-\boldsymbol{E}_{21})}{3!} + \frac{(ig_2)^4(\boldsymbol{E}_{11}+\boldsymbol{E}_{22})}{4!} + \frac{(g_2)^5(i)^4(\boldsymbol{E}_{12}-\boldsymbol{E}_{21})}{5!} \dots$$

This can be rearranged:

$$\exp(g_2\boldsymbol{X}_2) = \left(1 - \frac{(g_2)^2}{2!} + \frac{(g_2)^4}{4!} \dots\right)(\boldsymbol{E}_{11}+\boldsymbol{E}_{22}) + \left(g_2 - \frac{(g_2)^3}{3!} + \frac{(g_2)^5}{5!} \dots\right)(\boldsymbol{E}_{12}-\boldsymbol{E}_{21})$$

Therefore, $\exp(g_2\boldsymbol{X}_2)$ can be written out as

$$\exp(g_2\boldsymbol{X}_2) = \big(\cos(g_2)(\boldsymbol{E}_{11}+\boldsymbol{E}_{22}) + \sin(g_2)(\boldsymbol{E}_{12}-\boldsymbol{E}_{21})\big) = \begin{pmatrix} \cos(g_2) & \sin(g_2) \\ -\sin(g_2) & \cos(g_2) \end{pmatrix} \quad (S12)$$

Similarly, $\exp(g_3\boldsymbol{X}_3)$ can be expanded out as a series

$$\exp(g_3\boldsymbol{X}_3) = \boldsymbol{I} + g_3\boldsymbol{X}_3 + \frac{(g_3\boldsymbol{X}_3)^2}{2!} + \frac{(g_3\boldsymbol{X}_3)^3}{3!} + \frac{(g_3\boldsymbol{X}_3)^4}{4!} + \frac{(g_3\boldsymbol{X}_3)^5}{5!} \dots$$

Using

$$(\boldsymbol{X}_3)^\beta = \begin{cases} (i)^\beta(\boldsymbol{E}_{11}-\boldsymbol{E}_{22}) & \text{for } \beta \in \{1,3,5,7 \dots\} \\ (i)^\beta(\boldsymbol{E}_{11}+\boldsymbol{E}_{22}) & \text{for } \beta \in \{2,4,6,8 \dots\} \end{cases}$$

And $\boldsymbol{I} = \boldsymbol{E}_{11}+\boldsymbol{E}_{22}$, substituting these relations into the $\exp(g_3\boldsymbol{X}_3)$ series gives:

$$\exp(g_3\boldsymbol{X}_3) = (\boldsymbol{E}_{11}+\boldsymbol{E}_{22}) + ig_3(\boldsymbol{E}_{12}-\boldsymbol{E}_{21}) + \frac{(ig_3)^2(\boldsymbol{E}_{11}+\boldsymbol{E}_{22})}{2!} + \frac{(ig_3)^3(\boldsymbol{E}_{12}-\boldsymbol{E}_{21})}{3!} + \frac{(ig_3)^4(\boldsymbol{E}_{11}+\boldsymbol{E}_{22})}{4!} + \frac{(ig_3)^5(\boldsymbol{E}_{12}-\boldsymbol{E}_{21})}{5!} \dots$$

This can be rearranged:

$$\exp(g_3\boldsymbol{X}_3) = \left(1 + ig_3 + \frac{(ig_3)^2}{2!} + \frac{(ig_3)^3}{3!} + \frac{(ig_3)^4}{4!} + \frac{(ig_3)^5}{5!} \dots\right)\boldsymbol{E}_{11} + \left(1 - ig_3 + \frac{(ig_3)^2}{2!} - \frac{(ig_3)^3}{3!} + \frac{(ig_3)^4}{4!} - \frac{(ig_3)^5}{5!} \dots\right)\boldsymbol{E}_{22}$$

Therefore, $\exp(g_3\boldsymbol{X}_3)$ can be written out

$$\exp(g_3\boldsymbol{X}_3) = \left(e^{ig_3}\boldsymbol{E}_{11} + e^{-ig_3}\boldsymbol{E}_{22}\right) = \begin{pmatrix} e^{ig_3} & 0 \\ 0 & e^{-ig_3} \end{pmatrix} \quad (S13)$$

Multiplying equations $(S10)$, $(S11)$ and $(S12)$

$$\boldsymbol{U} = \begin{pmatrix} \cos(g_1) & i\sin(g_1) \\ i\sin(g_1) & \cos(g_1) \end{pmatrix} \cdot \begin{pmatrix} \cos(g_2) & \sin(g_2) \\ -\sin(g_2) & \cos(g_2) \end{pmatrix} \cdot \begin{pmatrix} e^{ig_3} & 0 \\ 0 & e^{-ig_3} \end{pmatrix}$$

This leads to equation (24) of the main text.

$$\boldsymbol{U} = \begin{pmatrix} e^{ig_3}\big(\cos(g_1)\cos(g_2) - i\sin(g_1)\sin(g_2)\big) & e^{-ig_3}\big(\cos(g_1)\sin(g_2) + i\sin(g_1)\cos(g_2)\big) \\ -e^{ig_3}\big(\cos(g_1)\sin(g_2) - i\sin(g_1)\cos(g_2)\big) & e^{-ig_3}\big(\cos(g_1)\cos(g_2) + i\sin(g_1)\sin(g_2)\big) \end{pmatrix} \quad (S14)$$

Each factor of $\boldsymbol{U}$ (equations $(S10)$, $(S11)$ and $(S12)$) is a square matrix $\exp(g_k\boldsymbol{X}_k) \equiv \boldsymbol{R}_k$ with the following properties: Property 1: $\det(\boldsymbol{R}_k) = 1$; and Property 2: $\boldsymbol{R}_k{}^{-1} = \boldsymbol{R}_k{}^{\dagger}$. Both of these

points can be proven analytically, without the need for numerical values of the $\{g_i\}$. For the two state system this is done here explicitly.

Proof of property 1: $\det(\boldsymbol{R}_k) = 1$

$$\det(\boldsymbol{R}_1) = \begin{vmatrix} \cos(g_1) & i\sin(g_1) \\ i\sin(g_1) & \cos(g_1) \end{vmatrix} = \left(\cos(g_1)\right)^2 - \left(i\sin(g_1)\right)^2 = \cos^2(g_1) + \sin^2(g_1) = 1$$

$$\det(\boldsymbol{R}_2) = \begin{vmatrix} \cos(g_2) & \sin(g_2) \\ -\sin(g_2) & \cos(g_2) \end{vmatrix} = \cos^2(g_1) - \left(-\sin^2(g_1)\right) = \cos^2(g_1) + \sin^2(g_1) = 1$$

$$\det(\boldsymbol{R}_3) = \begin{vmatrix} e^{ig_3} & 0 \\ 0 & e^{-ig_3} \end{vmatrix} = e^{ig_3}e^{-ig_3} = 1$$

Proof of property 2: ${\boldsymbol{R}_k}^{-1} = {\boldsymbol{R}_k}^{\dagger}$

$${\boldsymbol{R}_k}^{-1} = \frac{1}{\det(\boldsymbol{R}_k)} adj(\boldsymbol{R}_k)$$

As, according to proposition 1, $\det(\boldsymbol{R}_k) = 1 \; \forall \; k$,

$${\boldsymbol{R}_1}^{-1} = \mathrm{adj}(\boldsymbol{R}_1) = \begin{pmatrix} \cos(g_1) & -i\sin(g_1) \\ -i\sin(g_1) & \cos(g_1) \end{pmatrix} = {\boldsymbol{R}_1}^{\dagger}$$

$${\boldsymbol{R}_2}^{-1} = \mathrm{adj}(\boldsymbol{R}_2) = \begin{pmatrix} \cos(g_2) & -\sin(g_2) \\ \sin(g_2) & \cos(g_2) \end{pmatrix} = {\boldsymbol{R}_2}^{\dagger}$$

$${\boldsymbol{R}_3}^{-1} = \mathrm{adj}(\boldsymbol{R}_3) = \begin{pmatrix} e^{-ig_3} & 0 \\ 0 & e^{ig_3} \end{pmatrix} = {\boldsymbol{R}_3}^{\dagger}$$

Properties 1 and 2 mean that the $\boldsymbol{R}_k$ are rotation matrices, and as the product of two rotation matrices is always equal to a rotation matrix, $\boldsymbol{U} = \prod_k \boldsymbol{R}_k$ is also a rotation matrix.

As above

$$\boldsymbol{U}^{-1} = \exp(-g_3\boldsymbol{X}_3)\exp(-g_2\boldsymbol{X}_2)\exp(-g_1\boldsymbol{X}_1)$$

can be likewise found

$$\boldsymbol{U}^{-1} = \begin{pmatrix} e^{-ig_3}\left(\cos(g_1)\cos(g_2) + i\sin(g_1)\sin(g_2)\right) & -e^{-ig_3}\left(\cos(g_1)\sin(g_2) + i\sin(g_1)\cos(g_2)\right) \\ e^{ig_3}\left(\cos(g_1)\sin(g_2) - i\sin(g_1)\cos(g_2)\right) & e^{ig_3}\left(\cos(g_1)\cos(g_2) - i\sin(g_1)\sin(g_2)\right) \end{pmatrix} \quad (S15)$$

Alternatively, equation $(S15)$ and be found by inversion of equation $(S14)$. $\boldsymbol{U}$ is unitary, and as such satisfies $\boldsymbol{U}^{-1} = \boldsymbol{U}^{\dagger}$.

1.3 Evolution of the two state operators in Heisenberg picture

The operators, the $\boldsymbol{X}_k$, can be propagated using the Heisenberg picture:

$$\boldsymbol{X}(t) = \boldsymbol{U}^{-1}\boldsymbol{X}(0)\boldsymbol{U}$$

Using the above $\boldsymbol{U}$ and $\boldsymbol{U}^{-1}$ (equations $(S14)$ and $(S15)$), operators in the Heisenberg picture $\boldsymbol{X}_k(t)$ can be calculated. As noted in the main text, operators in the Heisenberg picture are the

adjoints of the dynamical symmetries $\boldsymbol{X}_k(t) = \boldsymbol{\mathcal{X}}_k\,(-t)$. The objective of this derivation is to produce a time correlation matrix from the Schrödinger to the Heisenberg picture, $\boldsymbol{B}(t)$, such that $\langle \boldsymbol{X}_k(t) \rangle$ can be acquired using Equation (35) of the main text:

$$\overline{\mathbf{X}}(t) = \boldsymbol{B}(t)\overline{\mathbf{X}}(0)$$

Where $\overline{\mathbf{X}}(t)$ is not an operator but a the vector of all the expectation values (at the time *t*) of the Heisenberg operators $\langle \boldsymbol{X}_k(t) \rangle$ and $\overline{\mathbf{X}}(0)$ is the vector of the expectation values at time t=0 $\langle \boldsymbol{X}_k(0) \rangle$.

For the two state case, equati

$$\boldsymbol{U} = \begin{pmatrix} e^{ig_3}\big(\cos(g_1)\cos(g_2) - i\sin(g_1)\sin(g_2)\big) & e^{-ig_3}\big(\cos(g_1)\sin(g_2) + i\sin(g_1)\cos(g_2)\big) \\ -e^{ig_3}\big(\cos(g_1)\sin(g_2) - i\sin(g_1)\cos(g_2)\big) & e^{-ig_3}\big(\cos(g_1)\cos(g_2) + i\sin(g_1)\sin(g_2)\big) \end{pmatrix}$$

on $(S21)$ can be written out in full

$$\begin{pmatrix} \langle \boldsymbol{X}_1(t) \rangle \\ \langle \boldsymbol{X}_2(t) \rangle \\ \langle \boldsymbol{X}_3(t) \rangle \end{pmatrix} = \begin{pmatrix} b_{11} & b_{12} & b_{13} \\ b_{21} & b_{22} & b_{23} \\ b_{31} & b_{32} & b_{33} \end{pmatrix} \cdot \begin{pmatrix} \langle \boldsymbol{X}_1(0) \rangle \\ \langle \boldsymbol{X}_2(0) \rangle \\ \langle \boldsymbol{X}_3(0) \rangle \end{pmatrix} \qquad \text{(S16)}$$

Equation (S15) is the matrix form of a set of linear equations, $\langle \boldsymbol{X}_k(t) \rangle = \sum_j b_{kj}\,\langle \boldsymbol{X}_k(0) \rangle$, each of which is calculable from $\boldsymbol{X}_k(t) = \boldsymbol{U}^{-1}\boldsymbol{X}_k(0)\boldsymbol{U}$ using the matrices of the evolution operator derived in Section 1.2 above.

Taking the two state system to be initially in a pure state

$$\boldsymbol{\rho}(0) = \begin{pmatrix} 1 & 0 \\ 0 & 0 \end{pmatrix}$$

$\langle \boldsymbol{X}_k(t) \rangle = Tr(\boldsymbol{\rho}(t) \cdot \boldsymbol{X}_k)$ therefore$\langle \boldsymbol{X}_k(0) \rangle = Tr(\boldsymbol{\rho}(0) \cdot \boldsymbol{X}_k)$. Because $Tr(\boldsymbol{\rho}(0) \cdot \boldsymbol{X}_1) = Tr(\boldsymbol{\rho}(0) \cdot \boldsymbol{X}_2) = 0$, and therefore

$\langle \boldsymbol{X}_1(0) \rangle = \langle \boldsymbol{X}_2(0) \rangle = 0$

Because $Tr(\boldsymbol{\rho}(0) \cdot \boldsymbol{X}_3) = \ i$, therefore

$\langle \boldsymbol{X}_3(0) \rangle = i$

Collecting the results as a matrix equation, the explicit two state case of equation $(S16)$ becomes

$$\begin{pmatrix} \langle \boldsymbol{X}_1(t) \rangle \\ \langle \boldsymbol{X}_2(t) \rangle \\ \langle \boldsymbol{X}_3(t) \rangle \end{pmatrix} = \begin{pmatrix} b_{11} & b_{12} & b_{13} \\ b_{21} & b_{22} & b_{23} \\ b_{31} & b_{32} & b_{33} \end{pmatrix} \cdot \begin{pmatrix} 0 \\ 0 \\ i \end{pmatrix}$$

The expressions of the b_{ij} of equation $(S16)$ can be found by

$\boldsymbol{X}_1(t) = \boldsymbol{U}^{-1}\boldsymbol{X}_1\boldsymbol{U}$

$\boldsymbol{X}_1(t) =$

$$\begin{pmatrix} e^{-ig_3}\big(\cos(g_1)\cos(g_2) + i\sin(g_1)\sin(g_2)\big) & -e^{-ig_3}\big(\cos(g_1)\sin(g_2) + i\sin(g_1)\cos(g_2)\big) \\ e^{ig_3}\big(\cos(g_1)\sin(g_2) - i\sin(g_1)\cos(g_2)\big) & e^{ig_3}\big(\cos(g_1)\cos(g_2) - i\sin(g_1)\sin(g_2)\big) \end{pmatrix} \cdot$$

$$\begin{pmatrix} 0 & i \\ i & 0 \end{pmatrix} \cdot$$

$$\begin{pmatrix} e^{ig_3}\big(\cos(g_1)\cos(g_2) - i\sin(g_1)\sin(g_2)\big) & e^{-ig_3}\big(\cos(g_1)\sin(g_2) + i\sin(g_1)\cos(g_2)\big) \\ -e^{ig_3}\big(\cos(g_1)\sin(g_2) - i\sin(g_1)\cos(g_2)\big) & e^{-ig_3}\big(\cos(g_1)\cos(g_2) + i\sin(g_1)\sin(g_2)\big) \end{pmatrix}$$

Multiplying through gives

$$\boldsymbol{X}_1(t) = \begin{pmatrix} -i\sin(2g_2) & ie^{-2ig_3}\cos(2g_2) \\ ie^{2ig_3}\cos(2g_2) & i\sin(2g_2) \end{pmatrix}$$

Therefore

$$\boldsymbol{X}_1(\mathrm{t}) = \cos(2\mathrm{g}_3)\cos(2\mathrm{g}_2)\,\boldsymbol{X}_1 + \sin(2\mathrm{g}_3)\cos(2\mathrm{g}_2)\,\boldsymbol{X}_2 - \sin(2\mathrm{g}_2)\,\boldsymbol{X}_3 \qquad (S17)$$

Similarly

$$\boldsymbol{X}_2(t) = \begin{pmatrix} i\sin(2g_1)\cos(2g_2) & {x_{12}}^2 \\ {x_{21}}^2 & -i\sin(2g_1)\cos(2g_2) \end{pmatrix}$$

$${x_{12}}^2 = e^{-2ig_3}(\cos^2(g_1) - \sin^2(g_1) + i\sin(2g_1)\sin(2g_2))$$

$${x_{21}}^2 = e^{2ig_3}(-\cos^2(g_1) + \sin^2(g_1) + i\sin(2g_1)\sin(2g_2))$$

Therefore

$$\begin{aligned} \boldsymbol{X}_2(t) = \quad & (\sin(2g_1)\sin(2g_2)\cos(2g_3) + (\sin^2(g_1) - \cos^2(g_1))\sin(2g_3))\boldsymbol{X}_1 \\ & +(\sin(2g_1)\sin(2g_2)\sin(2g_3) + (\cos^2(g_1) - \sin^2(g_1))\cos(2g_3))\boldsymbol{X}_2 \\ & + \sin(2g_1)\cos(2g_2)\,\boldsymbol{X}_3 \end{aligned} \qquad (S18)$$

And finally

$$\boldsymbol{X}_3(t) = \begin{pmatrix} i\cos(2g_1)\cos(2g_2) & {x_{12}}^3 \\ {x_{21}}^3 & -i\cos(2g_1)\cos(2g_2) \end{pmatrix}$$

$${x_{12}}^3 = -2e^{-2ig_3}(\sin(g_1)\cos(g_2) - i\cos(g_1)\sin(g_2)) \cdot (\cos(g_1)\cos(g_2) + i\sin(g_1)\sin(g_2))$$

$${x_{21}}^3 = 2e^{2ig_3}(\sin(g_1)\cos(g_2) + i\cos(g_1)\sin(g_2)) \cdot (\cos(g_1)\cos(g_2) - i\sin(g_1)\sin(g_2))$$

Therefore

$$\begin{aligned} \boldsymbol{X}_3(t) = & (\sin(2g_1)\sin(2g_3) + \cos(2g_1)\sin(2g_2)\cos(2g_3))\boldsymbol{X}_1 \\ & -(\sin(2g_1)\cos(2g_3) - \cos(2g_1)\sin(2g_2)\sin(2g_3))\boldsymbol{X}_2 \\ & + \cos(2g_1)\cos(2g_2)\,\boldsymbol{X}_3 \end{aligned} \qquad (S19)$$

Equations $(S16)$ to $(S18)$ for $\boldsymbol{X}_k(t)$ in terms of the $\boldsymbol{X}_k$ can be made into equations for $\langle \boldsymbol{X}_k(t)\rangle$ and $\langle \boldsymbol{X}_k(0)\rangle$**.** This is by multiplying both by on the left by $\langle\psi(0)|$, and on the right by $|\psi(0)\rangle$. As $\boldsymbol{U}(0)=\boldsymbol{I}$, $\langle\psi(0)|\boldsymbol{X}_k\,|\psi(0)\rangle = \langle\psi(0)|\boldsymbol{U}^{-1}(0)\boldsymbol{X}_k\,\boldsymbol{U}(0)|\psi(0)\rangle = \langle \boldsymbol{X}_k(0)\rangle$, therefore

$$\langle \boldsymbol{X}_1(t)\rangle \ = \cos(2g_3)\cos(2g_2)\langle \boldsymbol{X}_1(0)\rangle + \sin(2g_3)\cos(2g_2)\langle \boldsymbol{X}_2(0)\rangle - \sin(2g_2)\langle \boldsymbol{X}_3(0)\rangle$$

$$\begin{aligned}\langle \boldsymbol{X}_2(t)\rangle = \quad & (\sin(2g_1)\sin(2g_2)\cos(2g_3) + (\sin^2(g_1)-\cos^2(g_1))\sin(2g_3))\langle \boldsymbol{X}_1(0)\rangle \\ & +(\sin(2g_1)\sin(2g_2)\sin(2g_3) + (\cos^2(g_1)-\sin^2(g_1))\cos(2g_3))\langle \boldsymbol{X}_2(0)\rangle \\ & +\sin(2g_1)\cos(2g_2)\langle \boldsymbol{X}_3(0)\rangle\end{aligned}$$

And

$$\begin{aligned}\langle \boldsymbol{X}_3(t)\rangle \ = & (\sin(2g_1)\sin(2g_3) + \cos(2g_1)\sin(2g_2)\cos(2g_3))\langle \boldsymbol{X}_1(0)\rangle \\ & -(\sin(2g_1)\cos(2g_3) - \cos(2g_1)\sin(2g_2)\sin(2g_3))\langle \boldsymbol{X}_2(0)\rangle \\ & +\cos(2g_1)\cos(2g_2)\langle \boldsymbol{X}_3(0)\rangle\end{aligned}$$

From these equations one can construct the time correlation matrix.

$$\boldsymbol{B} = \begin{pmatrix} b_{11} & b_{12} & -\sin(2g_2) \\ b_{21} & b_{22} & \sin(2g_1)\cos(2g_2) \\ b_{31} & b_{32} & \cos(2g_1)\cos(2g_2)\end{pmatrix}$$

where

$$\begin{aligned} b_{11} &= \cos(2g_2)\cos(2g_3) \\ b_{12} &= \cos(2g_2)\sin(2g_3) \\ b_{21} &= \sin(2g_1)\sin(2g_2)\cos(2g_3) - \cos(2g_1)\sin(2g_3) \\ b_{22} &= \sin(2g_1)\sin(2g_2)\sin(2g_3) + \cos(2g_1)\cos(2g_3) \\ b_{31} &= \cos(2g_1)\sin(2g_2)\cos(2g_3) + \sin(2g_1)\sin(2g_3) \\ b_{32} &= \cos(2g_1)\sin(2g_2)\sin(2g_3) - \sin(2g_1)\cos(2g_3)\end{aligned} \qquad (S20)$$

Note, a useful alternative derivation of the Heisenberg operators is possible using equation $(S5)$ to $(S8)$ in

$\boldsymbol{X}_k(t) = \boldsymbol{U}^{-1}\boldsymbol{X}_k\boldsymbol{U}$

$\boldsymbol{X}_k(t) = \left(\prod_{j=\nu}^{1}\exp\left(-g_j(t)\boldsymbol{X}_j\right)\right)\boldsymbol{X}_k\left(\prod_{j=1}^{\nu}\exp(g_j(t)\boldsymbol{X}_j)\right)$

can be written as equation (35) of the main text

$$\boldsymbol{X}_k(t) = \left(\prod_{j=\nu}^{1}\exp\left(-g_j(t)ad\boldsymbol{X}_j\right)\right)\boldsymbol{X}_k$$

In the same way as the columns of the Ξ matrix were calculated one can therefore calculate the $\boldsymbol{B}$ matrix that propagates the $\boldsymbol{X}_k(t)$.

$\boldsymbol{X}_1(t) = \exp(-g_3(t)ad\boldsymbol{X}_3)\exp(-g_2(t)ad\boldsymbol{X}_2)\exp(-g_1(t)ad\boldsymbol{X}_1)\,\boldsymbol{X}_1$

becomes

$\boldsymbol{X}_1(t) = \cos(2g_2)\cos(2g_3)\,\boldsymbol{X}_1 + \cos(2g_2)\sin(2g_3)\,\boldsymbol{X}_2 - \sin(2g_2)\,\boldsymbol{X}_3$

Similarly

$\boldsymbol{X}_2(t) = \exp(-g_3(t)ad\boldsymbol{X}_3)\exp(-g_2(t)ad\boldsymbol{X}_2)\exp(-g_1(t)ad\boldsymbol{X}_1)\,\boldsymbol{X}_2$

becomes

$\boldsymbol{X}_2(t) = (\sin(2g_1)\sin(2g_2)\cos(2g_3) - \cos(2g_1)\sin(2g_3))\boldsymbol{X}_1 + (\cos(2g_1)\cos(2g_3) + \sin(2g_1)\sin(2g_2)\sin(2g_3))\boldsymbol{X}_2 + \sin(2g_1)\cos(2g_2)\,\boldsymbol{X}_3$

And, finally

$\boldsymbol{X}_3(t) = \exp(-g_3(t)ad\boldsymbol{X}_3)\exp(-g_2(t)ad\boldsymbol{X}_2)\exp(-g_1(t)ad\boldsymbol{X}_1)\,\boldsymbol{X}_3$

becomes

$\boldsymbol{X}_3(t) = (\sin(2g_1)\sin(2g_3) + \cos(2g_1)\sin(2g_2)\cos(2g_3))\boldsymbol{X}_1 + (\cos(2g_1)\sin(2g_2)\sin(2g_3) - \sin(2g_1)\cos(2g_3))\boldsymbol{X}_2 + \cos(2g_1)\cos(2g_2)\,\boldsymbol{X}_3$

Thereby recovering equations $(S16)$, $(S17)$ and $(S18)$.

This latter method will be used to calculate numerically the **B** matrix that propagates the $\boldsymbol{X}_k(t)$ of larger systems.

1.4 Evolution of the two state operators in dynamical symmetry picture

The form of the dynamical symmetry operators is

$\boldsymbol{\mathcal{X}}_k(t) = \boldsymbol{U}\boldsymbol{X}_k\boldsymbol{U}^{-1}$

The expectation value, at the present time *t*, of the time dependent dynamical symmetry operator is

$$\langle \boldsymbol{\mathcal{X}}_k(t)\rangle = \langle\psi_0|\boldsymbol{U}^{-1}\boldsymbol{\mathcal{X}}_k\boldsymbol{U}|\psi_0\rangle = \langle\psi_0|\boldsymbol{U}^{-1}\boldsymbol{U}\boldsymbol{X}_k\boldsymbol{U}^{-1}\boldsymbol{U}|\psi_0\rangle = \langle\psi_0|\boldsymbol{X}_k|\psi_0\rangle = constant$$

This is equivalent to equation (5) of the main text

$$Tr\big(\boldsymbol{\rho}(t)\cdot\boldsymbol{\mathcal{X}}_k(\boldsymbol{t})\big) = Tr(\boldsymbol{U}(t)\boldsymbol{\rho}(0)\boldsymbol{U}^{-1}(t)\boldsymbol{U}\boldsymbol{X}_k\boldsymbol{U}^{-1}) = Tr(\boldsymbol{U}(t)\boldsymbol{\rho}(0)\boldsymbol{X}_k\boldsymbol{U}^{-1})$$
$$= Tr(\boldsymbol{U}^{-1}\boldsymbol{U}(t)\boldsymbol{\rho}(0)\boldsymbol{X}_k) = Tr(\boldsymbol{\rho}(0)\boldsymbol{X}_k) = constant$$

We show this result explicitly for the three dynamical symmetries

$$\boldsymbol{\rho}(t)\cdot\boldsymbol{\mathcal{X}}_{\boldsymbol{1}}(t) = \begin{pmatrix}\rho_{11} & \rho_{12}\\ \rho_{21} & \rho_{22}\end{pmatrix}\begin{pmatrix}x_{11}{}^1 & x_{12}{}^1\\ x_{21}{}^1 & x_{22}{}^1\end{pmatrix} =$$

$$\begin{pmatrix}\frac{1}{2}e^{2ig_3}(\sin(2g_1) + i\cos(2g_1)\sin(2g_2)) & ie^{2ig_3}(\cos(g_1)\cos(g_2) - i\sin(g_1)\sin(g_2))^2\\ ie^{2ig_3}(\sin(g_1)\cos(g_2) + i\cos(g_1)\sin(g_2))^2 & -\frac{1}{2}e^{2ig_3}(\sin(2g_1) + i\cos(2g_1)\sin(2g_2))\end{pmatrix}$$

Therefore

$$Tr(\boldsymbol{\rho}(t)\cdot\boldsymbol{\mathcal{X}}_1(t)) = 0 \qquad (S21)$$

Similarly

$$\boldsymbol{\rho}(t)\cdot\boldsymbol{\mathcal{X}}_2(t)=\begin{pmatrix}\rho_{11} & \rho_{12}\\ \rho_{21} & \rho_{22}\end{pmatrix}\begin{pmatrix}{x_{11}}^2 & {x_{12}}^2\\ {x_{21}}^2 & {x_{22}}^2\end{pmatrix}=$$

$$\begin{pmatrix}\frac{1}{2}e^{2ig_3}(\cos(2g_1)\sin(2g_2)-i\sin(2g_1)) & e^{2ig_3}(\cos(g_1)\cos(g_2)-i\sin(g_1)\sin(g_2))^2\\ e^{2ig_3}(\sin(g_1)\cos(g_2)+i\cos(g_1)\sin(g_2))^2 & -\frac{1}{2}e^{2ig_3}(\cos(2g_1)\sin(2g_2)-i\sin(2g_1))\end{pmatrix}$$

Therefore

$$Tr(\boldsymbol{\rho}(t)\cdot\boldsymbol{\mathcal{X}}_2(t))=0 \qquad (S22)$$

And finally

$$\boldsymbol{\rho}(t)\cdot\boldsymbol{\mathcal{X}}_3(t)=\begin{pmatrix}\rho_{11} & \rho_{12}\\ \rho_{21} & \rho_{22}\end{pmatrix}\begin{pmatrix}{x_{11}}^3 & {x_{12}}^3\\ {x_{21}}^3 & {x_{22}}^3\end{pmatrix}=$$

$$\begin{pmatrix}\frac{1}{4}i\big(2+\cos\big(2(g_1-g_2)\big)+\cos\big(2(g_1+g_2)\big)\big) & \frac{1}{2}(\sin(2g_1)\cos(2g_2)-i\sin(2g_2))\\ \frac{1}{2}(\sin(2g_1)\cos(2g_2)-i\sin(2g_2)) & -\frac{1}{4}i\big(-2+\cos\big(2(g_1-g_2)\big)+\cos\big(2(g_1+g_2)\big)\big)\end{pmatrix}$$

Therefore

$$Tr(\boldsymbol{\rho}(t)\cdot\boldsymbol{\mathcal{X}}_3(t))=i \qquad (S23)$$

The dynamical symmetries $\boldsymbol{\mathcal{X}}_k$, can be propagated from the Schrödinger picture using $\boldsymbol{\mathcal{X}}_k(t)=\boldsymbol{U}\boldsymbol{X}_k\boldsymbol{U}^{-1}$ which leads the set of linear equations $\boldsymbol{\mathcal{X}}_k=\sum_j a_{kj}\,\boldsymbol{X}_j$. The a_{kj} coefficients can be computed from the matrix form of the evolution operator, equations (S15) and (S16):

$$\boldsymbol{\mathcal{X}}_1(t)=$$

$$\begin{pmatrix}e^{ig_3}\big(\cos(g_1)\cos(g_2)-i\sin(g_1)\sin(g_2)\big) & e^{-ig_3}\big(\cos(g_1)\sin(g_2)+i\sin(g_1)\cos(g_2)\big)\\ -e^{ig_3}\big(\cos(g_1)\sin(g_2)-i\sin(g_1)\cos(g_2)\big) & e^{-ig_3}\big(\cos(g_1)\cos(g_2)+i\sin(g_1)\sin(g_2)\big)\end{pmatrix}\cdot$$

$$\begin{pmatrix}0 & i\\ i & 0\end{pmatrix}\cdot$$

$$\begin{pmatrix}e^{-ig_3}\big(\cos(g_1)\cos(g_2)+i\sin(g_1)\sin(g_2)\big) & -e^{-ig_3}\big(\cos(g_1)\sin(g_2)+i\sin(g_1)\cos(g_2)\big)\\ e^{ig_3}\big(\cos(g_1)\sin(g_2)-i\sin(g_1)\cos(g_2)\big) & e^{ig_3}\big(\cos(g_1)\cos(g_2)-i\sin(g_1)\sin(g_2)\big)\end{pmatrix}$$

Multiplying through gives

$$\boldsymbol{\mathcal{X}}_1(t)=\begin{pmatrix}{x_{11}}^1 & {x_{12}}^1\\ {x_{21}}^1 & {x_{22}}^1\end{pmatrix}$$

$${x_{11}}^1=i\big(\cos(2g_1)\sin(2g_2)\cos(2g_3)+\sin(2g_1)\sin(2g_3)\big)$$

$${x_{12}}^1=\big(\sin(2g_1)\sin(2g_2)\cos(2g_3)-\cos(2g_1)\sin(2g_3)\big)+i\cos(2g_2)\cos(2g_3)$$

$${x_{21}}^1=\big(-\sin(2g_1)\sin(2g_2)\cos(2g_3)+\cos(2g_1)\sin(2g_3)\big)+i\cos(2g_2)\cos(2g_3)$$

$${x_{22}}^1=-i\big(\cos(2g_1)\sin(2g_2)\cos(2g_3)+\sin(2g_1)\sin(2g_3)\big)$$

Therefore

$$\boldsymbol{\mathcal{X}}_1(t) = \cos(2g_2)\cos(2g_3)\boldsymbol{X}_1 \\ +\big(\sin(2g_1)\sin(2g_2)\cos(2g_3) - \cos(2g_1)\sin(2g_3)\big)\boldsymbol{X}_2 \\ +\big(\cos(2g_1)\sin(2g_2)\cos(2g_3) + \sin(2g_1)\sin(2g_3)\big)\boldsymbol{X}_3 \tag{S24}$$

Similarly

$$\boldsymbol{\mathcal{X}}_2(t) = \boldsymbol{U}\boldsymbol{X}_2\boldsymbol{U}^{-1}$$

$$\boldsymbol{\mathcal{X}}_2 = \begin{pmatrix} e^{ig_3}\big(\cos(g_1)\cos(g_2) - i\sin(g_1)\sin(g_2)\big) & e^{-ig_3}\big(\cos(g_1)\sin(g_2) + i\sin(g_1)\cos(g_2)\big) \\ -e^{ig_3}\big(\cos(g_1)\sin(g_2) - i\sin(g_1)\cos(g_2)\big) & e^{-ig_3}\big(\cos(g_1)\cos(g_2) + i\sin(g_1)\sin(g_2)\big) \end{pmatrix}$$

$$\cdot \begin{pmatrix} 0 & 1 \\ -1 & 0 \end{pmatrix}$$

$$\cdot \begin{pmatrix} e^{-ig_3}\big(\cos(g_1)\cos(g_2) + i\sin(g_1)\sin(g_2)\big) & -e^{-ig_3}\big(\cos(g_1)\sin(g_2) + i\sin(g_1)\cos(g_2)\big) \\ e^{ig_3}\big(\cos(g_1)\sin(g_2) - i\sin(g_1)\cos(g_2)\big) & e^{ig_3}\big(\cos(g_1)\cos(g_2) - i\sin(g_1)\sin(g_2)\big) \end{pmatrix}$$

Multiplying through gives

$$\boldsymbol{\mathcal{X}}_2(t) = \begin{pmatrix} {x_{11}}^2 & {x_{12}}^2 \\ {x_{21}}^2 & {x_{22}}^2 \end{pmatrix}$$

$${x_{11}}^2 = i\big(\cos(2g_1)\sin(2g_2)\sin(2g_3) - \sin(2g_1)\cos(2g_3)\big)$$

$${x_{12}}^2 = \big(\cos(2g_1)\cos(2g_3) + \sin(2g_1)\sin(2g_2)\sin(2g_3)\big) + i\cos(2g_2)\sin(2g_3)$$

$${x_{21}}^2 = -\big(\cos(2g_1)\cos(2g_3) + \sin(2g_1)\sin(2g_2)\sin(2g_3)\big) + i\cos(2g_2)\sin(2g_3)$$

$${x_{22}}^2 = -i\big(\cos(2g_1)\sin(2g_2)\sin(2g_3) - \sin(2g_1)\cos(2g_3)\big)$$

Therefore

$$\boldsymbol{\mathcal{X}}_2(t) = \cos(2g_2)\sin(2g_3)\boldsymbol{X}_1 \\ +\big(\cos(2g_1)\cos(2g_3) + \sin(2g_1)\sin(2g_2)\sin(2g_3)\big)\boldsymbol{X}_2 \\ +\big(\cos(2g_1)\sin(2g_2)\sin(2g_3) - \sin(2g_1)\cos(2g_3)\big)\boldsymbol{X}_3 \tag{S25}$$

And finally

$$\boldsymbol{\mathcal{X}}_3(t) = \boldsymbol{U}\boldsymbol{X}_3\boldsymbol{U}^{-1}$$

$$\boldsymbol{\mathcal{X}}_3 = \begin{pmatrix} e^{ig_3}\big(\cos(g_1)\cos(g_2) - i\sin(g_1)\sin(g_2)\big) & e^{-ig_3}\big(\cos(g_1)\sin(g_2) + i\sin(g_1)\cos(g_2)\big) \\ -e^{ig_3}\big(\cos(g_1)\sin(g_2) - i\sin(g_1)\cos(g_2)\big) & e^{-ig_3}\big(\cos(g_1)\cos(g_2) + i\sin(g_1)\sin(g_2)\big) \end{pmatrix}$$

$$\cdot \begin{pmatrix} i & 0 \\ 0 & -i \end{pmatrix}$$

$$\cdot \begin{pmatrix} e^{-ig_3}\big(\cos(g_1)\cos(g_2) + i\sin(g_1)\sin(g_2)\big) & -e^{-ig_3}\big(\cos(g_1)\sin(g_2) + i\sin(g_1)\cos(g_2)\big) \\ e^{ig_3}\big(\cos(g_1)\sin(g_2) - i\sin(g_1)\cos(g_2)\big) & e^{ig_3}\big(\cos(g_1)\cos(g_2) - i\sin(g_1)\sin(g_2)\big) \end{pmatrix}$$

Multiplying through gives

$$\boldsymbol{\mathcal{X}}_3(t) = \begin{pmatrix} i\cos(2g_1)\cos(2g_2) & {x_{12}}^3 \\ {x_{21}}^3 & -i\cos(2g_1)\cos(2g_2) \end{pmatrix}$$

$${x_{12}}^3 = \sin(2g_1)\cos(2g_2) - i\sin(2g_2)$$

$${x_{21}}^3 = -\sin(2g_1)\cos(2g_2) - i\sin(2g_2)$$

Therefore

$$\boldsymbol{\mathcal{X}}_3(t) = -\sin(2g_2)\boldsymbol{X}_1 + \sin(2g_1)\cos(2g_2)\boldsymbol{X}_2 + \cos(2g_1)\cos(2g_2)\boldsymbol{X}_3 \qquad (S26)$$

From these equations, on can construct a time correlation matrix for these dynamical symmetry operators

$$\boldsymbol{A}(t) = \begin{pmatrix} a_{11} & a_{12} & \cos(2g_1)\sin(2g_2)\cos(2g_3) + \sin(2g_1)sin(2g_3) \\ \alpha_{21} & a_{22} & \cos(2g_1)\sin(2g_2)\sin(2g_3) - \sin(2g_1)\cos(2g_3) \\ a_{31} & a_{32} & \cos(2g_1)\cos(2g_2) \end{pmatrix}$$

where

$$a_{11} = \cos(2g_2)\cos(2g_3)$$

$$a_{12} = \sin(2g_1)\sin(2g_2)\cos(2g_3) - \cos(2g_1)\sin(2g_3)$$

$$a_{21} = \cos(2g_2)\sin(2g_3)$$

$$a_{22} = \sin(2g_1)\sin(2g_2)\sin(2g_3) + \cos(2g_1)\cos(2g_3)$$

$$a_{31} = -\sin(2g_2)$$

$$a_{32} = \sin(2g_1)\cos(2g_2)$$

The time correlation matrix $\boldsymbol{A}(t)$ that relates the dynamical symmetries at the time t to their initial values is related to the time correlation matrix $\boldsymbol{B}(t)$ as defined above by

$$\boldsymbol{A}(t) = \boldsymbol{B}(t)^{-1}$$

As summarized in the perspective in the main text we use the elements of the matrix $\boldsymbol{A}(t)$ in the two dual roles that the dynamical symmetries play.

Section S2 : *N*-State Generalisation

S2.1 Evolution operator

The N-state system has a unitary evolution operator

$$\boldsymbol{U}(t) = \prod_{\boldsymbol{k=1}}^{\nu} \exp(g_k \boldsymbol{X}_k)$$

comprised of ν $\exp(g_k \mathbf{X}_k)$ segments. The system has an Altafini basis of η $SU(2)$ groups. The values of ν and η are given in table (1) of the main text for different values of N. We repeat it here for completeness

Table 1

N	$\nu = 3\eta$	$\eta = N(N-1)/2$
$N = 2$	$\nu = 3$	$\eta = 1$
$N = 3$	$\nu = 9$	$\eta = 3$
$N = 4$	$\nu = 18$	$\eta = 6$
$N = 5$	$\nu = 30$	$\eta = 10$
$N = 6$	$\nu = 45$	$\eta = 15$
$\vdots$	$\vdots$	$\vdots$

Each $SU(2)$ group is constituted of 3 skewed Hermitian generators

$$\begin{aligned} \boldsymbol{X}_a &= i(\boldsymbol{E}_{nm} + \boldsymbol{E}_{mn}) \\ \boldsymbol{X}_b &= (\boldsymbol{E}_{nm} - \boldsymbol{E}_{mn}) \\ \boldsymbol{X}_c &= i(\boldsymbol{E}_{nn} - \boldsymbol{E}_{mm}) \end{aligned} \qquad (S27)$$

The η $SU(2)$ groups correspond to the $\eta = N(N-1)/2$ combinations of $\{n \in 1, N-1; m \in n+1, N\}$.

The factors of $\mathbf{U}$ form derived in section S1 for the 2 state system can be generalized to η pairs of states. For the pair of states n and m there are three distinct factors, corresponding to the three generators in equation $(S27)$: $\exp(g_a \boldsymbol{X}_a)$; $\exp(g_b \boldsymbol{X}_b)$; and $\exp(g_c \boldsymbol{X}_c)$. The general form of each factor can be derived as follows. Beginning with a Taylor expansion of $\exp(g_a \boldsymbol{X}_a)$

$$\exp(g_a \boldsymbol{X}_a) = \boldsymbol{I} + g_a \boldsymbol{X}_a + \frac{(g_a \boldsymbol{X}_a)^2}{2!} + \frac{(g_a \boldsymbol{X}_a)^3}{3!} + \frac{(g_a \boldsymbol{X}_a)^4}{4!} + \frac{(g_a \boldsymbol{X}_a)^5}{5!} \dots$$

with

$$(\boldsymbol{X}_a)^{\beta} = \begin{cases} (i)^{\beta}(\boldsymbol{E}_{nm} + \boldsymbol{E}_{mn}) & for\ \beta \in \{1,3,5,7 \dots\} \\ (i)^{\beta}(\boldsymbol{E}_{nn} + \boldsymbol{E}_{mm}) & for\ \beta \in \{2,4,6,8 \dots\} \end{cases}$$

and $\boldsymbol{I} = \boldsymbol{E}_{nn} + \boldsymbol{E}_{mm} + \sum_{p \neq n, p \neq m}^{N} \boldsymbol{E}_{pp}$, the $\exp(g_a \boldsymbol{X}_a)$ series gives:

$\exp(g_a\boldsymbol{X}_a) = \boldsymbol{E}_{nn} + \boldsymbol{E}_{mm} + \sum_{p\neq n,m}^{N} \boldsymbol{E}_{pp} + ig_a(\boldsymbol{E}_{nm} + \boldsymbol{E}_{mn}) + \frac{(ig_a)^2(\boldsymbol{E}_{nn}+\boldsymbol{E}_{mm})}{2!} + \frac{(ig_a)^3(\boldsymbol{E}_{nm}+\boldsymbol{E}_{mn})}{3!} + \frac{(ig_a)^4(\boldsymbol{E}_{nn}+\boldsymbol{E}_{mm})}{4!} + \frac{(ig_a)^5(\boldsymbol{E}_{nm}+\boldsymbol{E}_{mn})}{5!} \ldots$

This can be rearranged:

$\exp(g_a\boldsymbol{X}_a) = \left(1 - \frac{{g_a}^2}{2!} + \frac{{g_a}^4}{4!} \ldots\right)(\boldsymbol{E}_{nn} + \boldsymbol{E}_{mm}) + i\left(g_a - \frac{{g_a}^3}{3!} + \frac{{g_a}^5}{5!} \ldots\right)(\boldsymbol{E}_{nm} + \boldsymbol{E}_{mn}) + \sum_{p\neq n,m}^{N} \boldsymbol{E}_{pp}$

Thereby $\exp(g_a\boldsymbol{X}_a)$ can be written out as a linear combination of $\boldsymbol{E}_{ij}$ and therefore as a matrix:

$$\exp(g_a\boldsymbol{X}_a) = \cos(g_a)(\boldsymbol{E}_{nn} + \boldsymbol{E}_{mm}) + i\sin(g_a)(\boldsymbol{E}_{nm} + \boldsymbol{E}_{mn}) + \sum_{p\neq n,m}^{\boldsymbol{N}} \boldsymbol{E}_{pp} \qquad (S28)$$

Similarly, $\exp(g_b\boldsymbol{X}_b)$ can be expanded out as a series

$\exp(g_b\boldsymbol{X}_b) = \boldsymbol{I} + g_b\boldsymbol{X}_b + \frac{(g_b\boldsymbol{X}_b)^2}{2!} + \frac{(g_b\boldsymbol{X}_b)^3}{3!} + \frac{(g_b\boldsymbol{X}_b)^4}{4!} + \frac{(g_b\boldsymbol{X}_b)^5}{5!} \ldots$

Using

$$(\boldsymbol{X}_b)^\beta = \begin{cases} (i)^{\beta-1}(\boldsymbol{E}_{nm} - \boldsymbol{E}_{mn}) & for\ \beta \in \{1,3,5,7 \ldots\} \\ (i)^{\beta}(\boldsymbol{E}_{nn} + \boldsymbol{E}_{mm}) & for\ \beta \in \{2,4,6,8 \ldots\} \end{cases}$$

And $\boldsymbol{I} = \boldsymbol{E}_{nn} + \boldsymbol{E}_{mm} + \sum_{p\neq n,m}^{N} \boldsymbol{E}_{pp}$, substituting these relations into the $\exp(g_b\boldsymbol{X}_b)$ series gives:

$\exp(g_b\boldsymbol{X}_b) = \boldsymbol{E}_{nn} + \boldsymbol{E}_{mm} + \sum_{p\neq n,m}^{N} \boldsymbol{E}_{pp} + g_b(\boldsymbol{E}_{nm} - \boldsymbol{E}_{mn}) + \frac{(ig_b)^2(\boldsymbol{E}_{nn}+\boldsymbol{E}_{mm})}{2!} + \frac{(g_b)^3(i)^2(\boldsymbol{E}_{nm}-\boldsymbol{E}_{mn})}{3!} + \frac{(ig_b)^4(\boldsymbol{E}_{nn}+\boldsymbol{E}_{mm})}{4!} + \frac{(g_b)^5(i)^4(\boldsymbol{E}_{nm}-\boldsymbol{E}_{mn})}{5!} \ldots$

This can be rearranged:

$\exp(g_b\boldsymbol{X}_b) = \left(1 - \frac{{g_b}^2}{2!} + \frac{{g_b}^4}{4!} \ldots\right)(\boldsymbol{E}_{nn} + \boldsymbol{E}_{mm}) + \left(g_b - \frac{{g_b}^3}{3!} + \frac{{g_b}^5}{5!} \ldots\right)(\boldsymbol{E}_{nm} - \boldsymbol{E}_{mn}) + \sum_{p\neq n,m}^{N} \boldsymbol{E}_{pp}$

Therefore, $\exp(g_b\boldsymbol{X}_b)$ can be written out as

$$\exp(g_b\boldsymbol{X}_b) = \cos(g_b)(\boldsymbol{E}_{nn} + \boldsymbol{E}_{mm}) + \sin(g_b)(\boldsymbol{E}_{nm} - \boldsymbol{E}_{mn}) + \sum_{p\neq n,m}^{N} \boldsymbol{E}_{pp} \qquad (S29)$$

Similarly, $\exp(g_c\boldsymbol{X}_c)$ can be expanded out as a series

$\exp(g_c\boldsymbol{X}_c) = \boldsymbol{I} + g_c\boldsymbol{X}_c + \frac{(g_c\boldsymbol{X}_c)^2}{2!} + \frac{(g_c\boldsymbol{X}_c)^3}{3!} + \frac{(g_c\boldsymbol{X}_c)^4}{4!} + \frac{(g_c\boldsymbol{X}_c)^5}{5!} \ldots$

Using

$$(\boldsymbol{X}_c)^\beta = \begin{cases} (i)^{\beta}(\boldsymbol{E}_{nn} - \boldsymbol{E}_{mm}) & for\ \beta \in \{1,3,5,7 \ldots\} \\ (i)^{\beta}(\boldsymbol{E}_{nn} + \boldsymbol{E}_{mm}) & for\ \beta \in \{2,4,6,8 \ldots\} \end{cases}$$

And $\boldsymbol{I} = \boldsymbol{E}_{nn} + \boldsymbol{E}_{mm} + \sum_{p\neq n,m}^{N} \boldsymbol{E}_{pp}$, substituting these relations into the $\exp(g_c\boldsymbol{X}_c)$ series gives:

$\exp(g_c\boldsymbol{X}_c) = \boldsymbol{E}_{nn} + \boldsymbol{E}_{mm} + \sum_{p\neq n,m}^{N} \boldsymbol{E}_{pp} + ig_c(\boldsymbol{E}_{nn} - \boldsymbol{E}_{mm}) + \frac{(ig_c)^2(\boldsymbol{E}_{nn}+\boldsymbol{E}_{mm})}{2!} + \frac{(ig_c)^3(\boldsymbol{E}_{nn}-\boldsymbol{E}_{mm})}{3!} + \frac{(ig_c)^4(\boldsymbol{E}_{nn}+\boldsymbol{E}_{mm})}{4!} + \frac{(ig_c)^5(\boldsymbol{E}_{nn}-\boldsymbol{E}_{mm})}{5!} \dots$

This can be rearranged:

$\exp(g_c\boldsymbol{X}_c) = \left(1 + ig_c + \frac{(ig_c)^2}{2!} + \frac{(ig_c)^3}{3!} + \frac{(ig_c)^4}{4!} + \frac{(ig_c)^5}{5!} \dots\right)\boldsymbol{E}_{nn} + \left(1 - ig_c + \frac{(ig_c)^2}{2!} - \frac{(ig_c)^3}{3!} + \frac{(ig_c)^4}{4!} - \frac{(ig_c)^5}{5!} \dots\right)\boldsymbol{E}_{mm} + \sum_{p\neq n,m}^{N} \boldsymbol{E}_{pp}$

Therefore, $\exp(g_c\boldsymbol{X}_c)$ can be written out

$$\exp(g_c\boldsymbol{X}_c) = e^{ig_c}\boldsymbol{E}_{nn} + e^{-ig_c}\boldsymbol{E}_{mm} + \sum_{p\neq n,m}^{N} \boldsymbol{E}_{pp} \qquad (S30)$$

As all the Gelfand operators $\boldsymbol{E}_{nm}$ are square N dimensional matrices, $\boldsymbol{R}_k \equiv \exp(g_k\boldsymbol{X}_k)$ are similarly square matrices. It can be shown that equations $(S29)$, $(S30)$ and

$$\boldsymbol{U} = \begin{pmatrix} e^{ig_3}\big(\cos(g_1)\cos(g_2) - i\sin(g_1)\sin(g_2)\big) & e^{-ig_3}\big(\cos(g_1)\sin(g_2) + i\sin(g_1)\cos(g_2)\big) \\ -e^{ig_3}\big(\cos(g_1)\sin(g_2) - i\sin(g_1)\cos(g_2)\big) & e^{-ig_3}\big(\cos(g_1)\cos(g_2) + i\sin(g_1)\sin(g_2)\big) \end{pmatrix}$$

(S31)

have the properties of rotation matrixes: $\det(\boldsymbol{R}_k) = 1$ and $\boldsymbol{R}_k^{-1} = \boldsymbol{R}_k^{\dagger}$.

Proof of property 1: $\det(\boldsymbol{R}_k) = 1$

Beginning with $\det(\boldsymbol{R}_k)$ for $k = a$ and b. $\det(\mathbf{R}_k)$ will only have two non-zero components. The first of these is the product of diagonal coefficients, $\alpha = \cos(g_k)\cos(g_k)\prod 1 = \cos^2(g_k)$. The second is the product of the coefficients of $\boldsymbol{E}_{nm}$ and $\boldsymbol{E}_{mn}$ with the diagonal elements of $\boldsymbol{E}_{pp}$ for $p \neq n$ or m, these will be $\beta_a = i\sin(g_a)i\sin(g_b)\prod 1 = -\sin^2(g_b)$ and $\beta_b = \sin(g_b)\big(-\sin(g_b)\big)\prod 1 = -\sin^2(g_b)$. All other components of the determinant will be zero. Because of the permutation of swapping the columns $\boldsymbol{E}_{nn} \to \boldsymbol{E}_{nm}$ and $\boldsymbol{E}_{mm} \to \boldsymbol{E}_{mn}$, $\det(\boldsymbol{R}_k) = \alpha - \beta_k = \cos^2(g_k) + \sin^2(g_k) = 1$.

$\det(\boldsymbol{R}_c)$ will only have one non-zero component, the product of diagonal coefficients, $\alpha = e^{ig_c}e^{-ig_c}\prod 1 = 1$. Therefore $\det(\boldsymbol{R}_c) = 1$.

Proof of property 2: $\boldsymbol{R}_k^{-1} = \boldsymbol{R}_k^{\dagger}$.

Beginning with $\boldsymbol{R}_k^{-1}$ for $k = a$ and b. The adjugate of $\boldsymbol{R}_k$, $\mathrm{adj}(\boldsymbol{R}_k)$, will have the effect of swapping the coefficients of $\boldsymbol{E}_{nn}$ and $\boldsymbol{E}_{mm}$, whilst leaving the rest of the diagonal unchanged. As these coefficients are identical in equations $(S28)$ and $(S29)$, this will leave the whole diagonal unchanged. The adjugate of $\boldsymbol{R}_k$ will swap and invert the sign of the coefficients of

$\boldsymbol{E}_{nm}$ and $\boldsymbol{E}_{mn}$. In equation $(S30)$, therefore $i\sin(g_a)(\boldsymbol{E}_{nm}+\boldsymbol{E}_{mn}) \rightarrow -i\sin(g_a)(\boldsymbol{E}_{nm}+\boldsymbol{E}_{mn})$, and in equation $(S31)$, $\sin(g_b)(\boldsymbol{E}_{nm}-\boldsymbol{E}_{mn}) \rightarrow \sin(g_b)(-\boldsymbol{E}_{nm}+\boldsymbol{E}_{mn})$. These transformations are complex conjugations. Since $\det(\boldsymbol{R}_k)=1$, therefore $\boldsymbol{R}_k^{-1}=\mathrm{adj}(\boldsymbol{R}_k)=\boldsymbol{R}_k^{\dagger}$ for $k=a$ and b.

Since $\boldsymbol{R}_c$ is diagonal, $\mathrm{adj}(\boldsymbol{R}_c)$ will simply have the effect of swapping the coefficients of $\boldsymbol{E}_{nn}$ and $\boldsymbol{E}_{mm}$. This transformation, $e^{ig_c} \leftrightarrow e^{-ig_c}$ is a complex conjugation, therefore since $\boldsymbol{R}_c$ is diagonal and $\det(\boldsymbol{R}_c)=1$, $\boldsymbol{R}_c^{-1}=\mathrm{adj}(\boldsymbol{R}_c)=\boldsymbol{R}_c^{\dagger}$.

These proofs cover all generators in equation $(S27)$, and therefore the factors of any $\boldsymbol{U}$ will be rotation matrices. By the fact that the product of rotation matrices is a rotation matrix, $\boldsymbol{U}=\prod_k \boldsymbol{R}_k$ will therefore always be a rotation matrix when constructed in this basis of generators.

S2.2 Constructing the Hamiltonian and h coefficient vector

The Hamiltonian of the N state system

$$\boldsymbol{H}(t)=\sum_{n,m}^{N} c_{nm}(t)\boldsymbol{E}_{nm} \tag{S32}$$

Where

$$c_{mn}(t)=\begin{cases}\alpha_n & for\ n=m\\ -E(t)\mu_{nm} & for\ n\neq m\end{cases} \tag{S33}$$

For example, for $N=3$

$$\boldsymbol{H}(t)=\begin{pmatrix}\alpha_1 & -E(t)\mu_{12} & -E(t)\mu_{13}\\ -E(t)\mu_{12} & \alpha_2 & -E(t)\mu_{23}\\ -E(t)\mu_{13} & -E(t)\mu_{23} & \alpha_3\end{pmatrix} \tag{S34}$$

Taking $\alpha_1 \equiv 0$, the α_n are the transition energies from the ground to the nth excited state, $n>1$. In the $\{\boldsymbol{X}_k\}$ basis the Hamiltonian of the N state system is

$$\boldsymbol{H}(t)=\sum_k^{\nu} h_k(t)\boldsymbol{X}_k \tag{S35}$$

Where there are three structures of $h_k(t)$

$$h_k(t)=\begin{cases} iE(t)\mu_{nm} & for\ Mod(k-1,3)=0\\ 0 & for\ Mod(k-2,3)=0\\ i\frac{1}{N}(-\alpha_n+\alpha_m) & for\ Mod(k,3)=0\end{cases} \tag{S36}$$

For the $\eta=N(N-1)/2$ combinations of states $\{n\ \in 1, N-1; m \in n+1, N\}$. For $N=3$, equation $(S33)$ is

$$\boldsymbol{H}(t)=iE(t)\mu_{12}\boldsymbol{X}_1+i\frac{1}{3}(-\alpha_1+\alpha_2)\boldsymbol{X}_3+iE(t)\mu_{13}\boldsymbol{X}_4+i\frac{1}{3}(-\alpha_1+\alpha_3)\boldsymbol{X}_6+iE(t)\mu_{23}\boldsymbol{X}_7+i\frac{1}{3}(-\alpha_2+\alpha_3)\boldsymbol{X}_9$$

The **h** vector of this Hamiltonian is

$$\mathbf{h} = \begin{pmatrix} iE(t)\mu_{12} \\ 0 \\ i\frac{1}{3}(-\alpha_1 + \alpha_2) \\ iE(t)\mu_{13} \\ 0 \\ i\frac{1}{3}(-\alpha_1 + \alpha_3) \\ iE(t)\mu_{23} \\ 0 \\ i\frac{1}{3}(-\alpha_2 + \alpha_3) \end{pmatrix}$$

S2.3 Constructing Ξ

The nth column of the Ξ matrix is

$\exp(g_1 ad\boldsymbol{X}_1)\exp(g_2 ad\boldsymbol{X}_2) \ldots \exp(g_{n-2} ad\boldsymbol{X}_{n-2})\exp(g_{n-1} ad\boldsymbol{X}_{n-1})\,\boldsymbol{X}_n$

Expanding out $\exp(g_m ad\boldsymbol{X}_m)\,\boldsymbol{X}_n$ with a Taylor series and using the commutator relations in table $(S3)$ below shows that

$$\exp(g_m ad\boldsymbol{X}_m)\,\boldsymbol{X}_n = \boldsymbol{X}_n + \frac{c_{m,n}}{c_{m,[m,n]}}\sin\left(c_{m,[m,n]}g_m\right)\boldsymbol{X}_{[m,n]} + \frac{c_{m,n}}{c_{m,[m,n]}}\left(1 - \cos\left(c_{m,[m,n]}g_m\right)\right)\boldsymbol{X}_{[m,[m,n]]} \qquad (S37)$$

Where c are structure constants, and the notations $c_{m,n}$ and $\boldsymbol{X}_{[m,n]}$ refer to

$$[\boldsymbol{X}_m, \boldsymbol{X}_n] = c_{m,n}\boldsymbol{X}_{[m,n]} \qquad (S38)$$

and $c_{m,[m,n]}$ and $\boldsymbol{X}_{[m,[m,n]]}$ refer to

$$\left[\boldsymbol{X}_m, [\boldsymbol{X}_m, \boldsymbol{X}_n]\right] = c_{m,[m,n]}\boldsymbol{X}_{[m,[m,n]]} \qquad (S39)$$

Hence, each $\exp(g_m ad\boldsymbol{X}_m)\,\boldsymbol{X}_n$ can be calculated using equation $(S35)$, taking the $c_{*,*}$ and $\boldsymbol{X}_{[*,*]}$ from the commutator relations. Table $(S3)$ is the $N = 3$ commutator table

Table S2: The commutation relations for the three state $\{\boldsymbol{X}_k\}$ generators

	$[\cdot,\boldsymbol{X}_1]$	$[\cdot,\boldsymbol{X}_2]$	$[\cdot,\boldsymbol{X}_3]$	$[\cdot,\boldsymbol{X}_4]$	$[\cdot,\boldsymbol{X}_5]$	$[\cdot,\boldsymbol{X}_6]$	$[\cdot,\boldsymbol{X}_7]$	$[\cdot,\boldsymbol{X}_8]$	$[\cdot,\boldsymbol{X}_9]$
$[\boldsymbol{X}_1,\cdot]$	0	$-2\boldsymbol{X}_3$	$2\boldsymbol{X}_2$	$-\boldsymbol{X}_8$	$\boldsymbol{X}_7$	$\boldsymbol{X}_2$	$-\boldsymbol{X}_5$	$\boldsymbol{X}_4$	$\boldsymbol{X}_2$
$[\boldsymbol{X}_2,\cdot]$	$2\boldsymbol{X}_3$	0	$-2\boldsymbol{X}_1$	$-\boldsymbol{X}_7$	$-\boldsymbol{X}_8$	$-\boldsymbol{X}_1$	$-\boldsymbol{X}_4$	$\boldsymbol{X}_5$	$\boldsymbol{X}_1$
$[\boldsymbol{X}_3,\cdot]$	$-2\boldsymbol{X}_2$	$2\boldsymbol{X}_1$	0	$-\boldsymbol{X}_5$	$\boldsymbol{X}_4$	0	$\boldsymbol{X}_8$	$-\boldsymbol{X}_7$	0
$[\boldsymbol{X}_4,\cdot]$	$\boldsymbol{X}_8$	$\boldsymbol{X}_7$	$\boldsymbol{X}_5$	0	$-2\boldsymbol{X}_6$	$2\boldsymbol{X}_5$	$-\boldsymbol{X}_2$	$-\boldsymbol{X}_1$	$\boldsymbol{X}_5$
$[\boldsymbol{X}_5,\cdot]$	$-\boldsymbol{X}_7$	$\boldsymbol{X}_8$	$-\boldsymbol{X}_4$	$2\boldsymbol{X}_6$	0	$-2\boldsymbol{X}_4$	$\boldsymbol{X}_1$	$-\boldsymbol{X}_2$	$-\boldsymbol{X}_4$
$[\boldsymbol{X}_6,\cdot]$	$-\boldsymbol{X}_2$	$\boldsymbol{X}_1$	0	$-2\boldsymbol{X}_5$	$2\boldsymbol{X}_4$	0	$-\boldsymbol{X}_8$	$\boldsymbol{X}_7$	0
$[\boldsymbol{X}_7,\cdot]$	$\boldsymbol{X}_5$	$-\boldsymbol{X}_4$	$-\boldsymbol{X}_8$	$\boldsymbol{X}_2$	$-\boldsymbol{X}_1$	$\boldsymbol{X}_8$	0	$-2\boldsymbol{X}_9$	$2\boldsymbol{X}_8$
$[\boldsymbol{X}_8,\cdot]$	$-\boldsymbol{X}_4$	$-\boldsymbol{X}_5$	$\boldsymbol{X}_7$	$\boldsymbol{X}_1$	$\boldsymbol{X}_2$	$-\boldsymbol{X}_7$	$2\boldsymbol{X}_9$	0	$-2\boldsymbol{X}_7$

$[\boldsymbol{X}_9,\cdot]$	$\boldsymbol{X}_2$	$-\boldsymbol{X}_1$	0	$-\boldsymbol{X}_5$	$\boldsymbol{X}_4$	0	$-2\boldsymbol{X}_8$	$2\boldsymbol{X}_7$	0

By recursive application of equation ($S36$), $\boldsymbol{\Xi}$ can be constructed

S2.3 Constructing the time correlation matrices for the Heisenberg operators and the dynamical symmetries

Equation ($S22$) is the matrix form of a set of linear equations, each of which is derivable from $\boldsymbol{X}_k(t) = \boldsymbol{U}^{-1}\boldsymbol{X}_k\boldsymbol{U} = \left(\prod_{j=\nu}^{1}\exp\left(-g_j(t)\boldsymbol{X}_j\right)\right)\boldsymbol{X}_k\left(\prod_{j=1}^{\nu}\exp\left(g_j(t)\boldsymbol{X}_j\right)\right)$. Using equations ($S4$) to (S7) to expand out this equation, each row of $\boldsymbol{B}$ can be written as

$$\boldsymbol{X}_k(t) = \left(\prod_{j=9}^{\nu}\exp\left(-g_j(t)ad\boldsymbol{X}_j\right)\right)\boldsymbol{X}_n = \sum_{m=1}^{\nu} b_{km}\boldsymbol{X}_m \qquad (S40)$$

Where b_{nm} are the elements of $\boldsymbol{B}$.

The elements of $\boldsymbol{A}$ can be similarly calculated. Writing $\boldsymbol{\mathcal{X}}_k(t) = \boldsymbol{U}\boldsymbol{X}_k\boldsymbol{U}^{-1} = \left(\prod_{j=1}^{\nu}\exp\left(g_j(t)\boldsymbol{X}_j\right)\right)\boldsymbol{X}_k\left(\prod_{j=\nu}^{1}\exp\left(-g_j(t)\boldsymbol{X}_j\right)\right)$, we get the rows of $\boldsymbol{A}$

$$\boldsymbol{\mathcal{X}}_k(t) = \left(\prod_{j=1}^{\nu}\exp\left(g_j(t)ad\boldsymbol{X}_j\right)\right)\boldsymbol{X}_k = \sum_{m=1}^{\nu} a_{km}\boldsymbol{X}_m \qquad (S41)$$

Thereby we have provided a practical route to the dynamical symmetries in the N state case where the relation of ν to N is in table 1 above

S3. CdSe nanoparticle model systems

We consider CdSe nanoparticles of diameter 2 to 3 nm as in our previous works.[6-11].

The electronic structure of a single NP is modelled with two hole and one electron one particle states. This leads to two exciton states, 1S and 2S, per nanoparticle (NP).[12,13] Therefore, the CdSe NP level structure is modelled as a three electronic state system constituted of a ground state and two excited states.

To create a more complex system, we include the spin orbit interactions between the orbital angular momentum and spin of the two holes, which leads to 4 exciton states : $1S_{3/2}$, $1S_{1/2}$, $2S_{3/2}$ and $2S_{1/2}$.[12,13] Dimers are assembled by covalently linking two NPs with a short, 0.5 nm in length, dithiopropane ligand.[8,9] Because each NP has the same mean size but is drawn from an ensemble of size dispersed colloidal NPs, the dimers are quasi-homodimers with slightly different energies of electronic states that are determined by their size. In the quasi-homodimers, the quasi-isoenergetic bands of each QD (the $1S_{3/2}$, $1S_{1/2}$, $2S_{3/2}$ and $2S_{1/2}$) are split by the Coulomb interdot interactions into a higher and a lower dimer band giving: $1S^L_{3/2}$, $1S^H_{3/2}$, $1S^L_{1/2}$, $1S^H_{1/2}$, $2S^L_{3/2}$, $2S^H_{3/2}$, $2S^L_{1/2}$ and $2S^H_{1/2}$. Therefore, the dimer electronic structure is modelled by a nine state electronic states, a ground state and eight excited states.

The energy levels and dipole transition moments used in these examples approximate the NP electronic structure used in refs.[10,11]. The mean diameter of the colloidal CdSe NP, $\bar{D}$ is taken to be 3 nm. For this value of $\bar{D}$, the energetic ordering of the bands of both monomers and dimers is as previously enumerated.

The monomer and dimer NPs considered in the following two sections will be optically excited by a sequence of three fs laser pulses, as in the experimental set-up of 2D electron spectroscopy experiments.[6,8]

S3.1 Three state model

Figure 1 of the main text shows the three state level structure. The Hamiltonian of the system is

$$\boldsymbol{H}(t) = \begin{pmatrix} 0 & -E(t)\mu_{1,2} & -E(t)\mu_{1,3} \\ -E(t)\mu_{1,2} & \alpha_2 & 0 \\ -E(t)\mu_{1,3} & 0 & \alpha_3 \end{pmatrix}$$

the transition energies and transition dipole moments are given in Tables $(S4)$ and $(S5)$.

Table S3: Ground to electronically excited state transition energies of the monomer three state system

α_i	Transition Energies
α_2	$2.2\ eV$
α_3	$2.8eV$

Table S4: Ground to electronically excited state, and excited state to excited state transition dipole moments of the monomer three state system

$\mu_{i,j}$	Transition Dipole Moments
$\mu_{1,2}$	1.27 D
$\mu_{1,3}$	1.27 D

Each pulse in the sequence of three pulses has the same time profile of the electric field $E(t)$,

$$E(t) = \sum_{j=1}^{3} E_0 \exp\left(-\left(t - t_j\right)^2 / 2\,\sigma^2\right) \cos(\omega_c t) \qquad (S40)$$

With identical parameters

$$E_0 = 0.07\ a.u., \sigma = 2.49\ fs\ ,\omega_c = 2.5\ eV$$

The values of t_j's are set to

$$t_1 = 14.5\ fs, t_2 = 29\ fs, t_3 = 43.5\ fs$$

The $\{g_i\}$ can be calculated by solving equation $(S3)$ with a $\boldsymbol{\Xi}$ constructed from equation $(S35)$ to $(S37)$. The time dependence of the nine g_i coefficients of the terms of the evolution operators **U** are plotted in figure 2 of the main text. Figure 2 a, b and c show g_i which are associated with coherence operators, and panel d shows the g_i which are associated with population difference operators.

The expectation values of the Heisenberg operators can be calculated using equation $(S38)$, with ***B*** constructed using equations $(S35)$ and $(S37)$ and these $\{g_i\}$. Their time dependence is plotted in Figure 3 of the main text. The analytical expressions of the matrix elements of the **B** matrix needed to propagate the mean values of the Heisenberg operators for an initial state that is the ground state, see equation (37) of the main text, are given in section 3.4 below.

S3.2 Nine state dimer model

Figure 3 of the main text shows a schematic representation of level structure of the nine state dimer system. The values of the transition energies and dipole transition moments of this system are given in tables $(S6)$.

Table S5: Ground to electronically excited state transition energies of the dimer nine state system

α_i	Transition Energies	α_i	Transition Energies
α_2	$2.286\ eV$	α_6	$2.747\ eV$
α_3	$2.449\ eV$	α_7	$2.895\ eV$

α_4	$2.550\ eV$	α_8	$2.975\ eV$
α_5	$2.693\ eV$	α_9	$3.198\ eV$

Table S6: Ground to electronically excited state transition dipole moments of the dimer nine state system

$\mu_{i,j}$	Transition Dipole Moments	$\mu_{i,j}$	Transition Dipole Moments
$\mu_{1,2}$	28.0 D	$\mu_{1,6}$	12.4 D
$\mu_{1,3}$	20.1 D	$\mu_{1,7}$	18.6 D
$\mu_{1,4}$	5.4 D	$\mu_{1,8}$	11.0 D
$\mu_{1,5}$	6.3 D	$\mu_{1,9}$	9.5 D

The same pulse, $E(t)$ (equation $(S40)$), is used for the nine state system for the three state system, with same t_i values and pulse parameters

$$E_0 = 0.0003\ a.u., \sigma = 2.49\ fs\ ,\omega_c = 2.45\ eV$$

The $\{g_i\}$ can be calculated solving equation $(S9)$ with a $\boldsymbol{\Xi}$ constructed from equation $(S35)$ to (S37). Six of the 108 g_i which constitute the $\boldsymbol{U}$ are presented in figure of the main text. Panels a, c, e and f show g_i which are associated with coherence operators, and panels b and d show g_i which are associated with population difference operators.

The expectation values of the Heisenberg operators can be calculated using equation $(S38)$, with $\boldsymbol{B}$ constructed using equations $(S35)$ and $(S37)$ and these $\{g_i\}$. Their time dependence is plotted in Figure 6 of the main text

Section 3.3 Elements of the three state system time correlation matrix $\boldsymbol{B}$ of the Heisenberg operators

The rows of the three state system matrix $\boldsymbol{B}(t)$ are given by
$\left(\prod_{m=9}^{1} \exp(-g_m(t) ad\boldsymbol{X}_m)\right)\boldsymbol{X}_n = \sum_{m=1}^{9} \alpha_{nm}\boldsymbol{X}_m$

As explained in the main text, because the initial state is the ground state, the initial values of $\langle \boldsymbol{X}_k(0)\rangle$ that are not zero are $< \boldsymbol{X}_3(0) >$ and $< \boldsymbol{X}_6(0) >$ and one need only to calculate the 3rd and 6th columns of the three state system $\boldsymbol{B}$, equation (37) of the main text. This means we are only interested in the b_{nm} for $m = \{3,6\}$.

Looking at table (S3), one can see that no $\exp(-g_m(t) ad\boldsymbol{X}_m)\boldsymbol{X}_n$ will contribute to the coefficients of $\boldsymbol{X}_3(t)$ or $\boldsymbol{X}_6(t)$ for $m > 5$ and $\forall\, n$. This is because none of the commutation relations $[\boldsymbol{X}_m, \boldsymbol{X}_n]$ produce $c\boldsymbol{X}_3(t)$ or $c\boldsymbol{X}_6(t)$ for $m > 5$ and $\forall\, n$.

Hence, to find α_{n3} and α_{n6} one need only calculate
$\left(\prod_{m=5}^{1} \exp(-g_m(t) ad\boldsymbol{X}_m)\right)\boldsymbol{X}_n = \sum_{m=1}^{5} \alpha_{nm}\boldsymbol{X}_m$.

First, to find b_{13} and b_{16}
$\boldsymbol{X}_1'(t) = \exp(-g_5 ad\boldsymbol{X}_5)\exp(-g_4 ad\boldsymbol{X}_4)\exp(-g_3 ad\boldsymbol{X}_3)\exp(-g_2 ad\boldsymbol{X}_2)\exp(-g_1 ad\boldsymbol{X}_1)\boldsymbol{X}_1$
Where $\boldsymbol{X}_1'(t)$ is the part of $\boldsymbol{X}_1(t)$ sufficient for finding α_{13} and α_{16}
Using
$\exp(-g_1 ad\boldsymbol{X}_1)\boldsymbol{X}_1 = \boldsymbol{X}_1$
gives
$\boldsymbol{X}_1'(t) = \exp(-g_5 ad\boldsymbol{X}_5)\exp(-g_4 ad\boldsymbol{X}_4)\exp(-g_3 ad\boldsymbol{X}_3)\exp(-g_2 ad\boldsymbol{X}_2)\boldsymbol{X}_1$
Using the relation
$\exp(-g_2 ad\boldsymbol{X}_2)\boldsymbol{X}_1 = (\cos(2g_2)\boldsymbol{X}_1 + \sin(-2g_2)\boldsymbol{X}_3)$
derived from equation (25) of the main text and the commutator relations on Table 2, gives
$\boldsymbol{X}_1'(t) = \exp(-g_5 ad\boldsymbol{X}_5)\exp(-g_4 ad\boldsymbol{X}_4)\exp(-g_3 ad\boldsymbol{X}_3)(\cos(2g_2)\boldsymbol{X}_1 + \sin(-2g_2)\boldsymbol{X}_3)$
which can be rearranged to
$\boldsymbol{X}_1'(t) = \exp(-g_5 ad\boldsymbol{X}_5)\exp(-g_4 ad\boldsymbol{X}_4)(\cos(2g_2)\exp(-g_3 ad\boldsymbol{X}_3)\boldsymbol{X}_1 +$
$\sin(-2g_2)\exp(-g_3 ad\boldsymbol{X}_3)\boldsymbol{X}_3)$
Using the similarly derived relations
$\exp(-g_3 ad\boldsymbol{X}_3)\boldsymbol{X}_1 = (\cos(2g_3)\boldsymbol{X}_1 + \sin(2g_3)\boldsymbol{X}_2)$,
$\exp(-g_3 ad\boldsymbol{X}_3)\boldsymbol{X}_3 = \boldsymbol{X}_3$

This becomes

$\boldsymbol{X}_1'(t) = \exp(-g_5 ad\boldsymbol{X}_5)(\cos(2g_2)\cos(2g_3)\exp(-g_4 ad\boldsymbol{X}_4)\boldsymbol{X}_1 +$

$\cos(2g_2)\sin(2g_3)\exp(-g_4 ad\boldsymbol{X}_4)\boldsymbol{X}_2 + \sin(-2g_2)\exp(-g_4 ad\boldsymbol{X}_4)\boldsymbol{X}_3)$

Using the similarly derived relations

$\exp(-g_4 ad\boldsymbol{X}_4)\boldsymbol{X}_3 = \left(\boldsymbol{X}_3 + \frac{1}{2}\sin(-2g_4)\boldsymbol{X}_5 + \frac{1}{2}\left(-1 + \cos(2g_4)\right)\boldsymbol{X}_6\right),$

$\exp(-g_4 ad\boldsymbol{X}_4)\boldsymbol{X}_2 = (\cos(g_4)\boldsymbol{X}_2 + \sin(-g_4)\boldsymbol{X}_7),$

$\exp(-g_4 ad\boldsymbol{X}_4)\boldsymbol{X}_1 = (\cos(g_4)\boldsymbol{X}_1 + \sin(-g_4)\boldsymbol{X}_8)$

gives

$\boldsymbol{X}_1'(t) = \cos(2g_2)\cos(2g_3)\cos(g_4)\exp(-g_5 ad\boldsymbol{X}_5)\boldsymbol{X}_1 +$

$\cos(2g_2)\sin(2g_3)\cos(g_4)\exp(-g_5 ad\boldsymbol{X}_5)\boldsymbol{X}_2 + \sin(-2g_2)\exp(-g_5 ad\boldsymbol{X}_5)\boldsymbol{X}_3 +$

$\frac{1}{2}\sin(2g_2)\sin(2g_4)\exp(-g_5 ad\boldsymbol{X}_5)\boldsymbol{X}_5 + \frac{1}{2}\sin(2g_2)\left(1 - \cos(2g_4)\right)\exp(-g_5 ad\boldsymbol{X}_5)\boldsymbol{X}_6 +$

$\cos(2g_2)\sin(2g_3)\sin(-g_4)\exp(-g_5 adX_5)\boldsymbol{X}_7 + \cos(2g_2)\cos(2g_3)\sin(-g_4)\exp(-g_5 ad\boldsymbol{X}_5)\boldsymbol{X}_8$

Neither $\exp(-g_5 ad\boldsymbol{X}_5)\boldsymbol{X}_1$, $\exp(-g_5 ad\boldsymbol{X}_5)\boldsymbol{X}_2$, $\exp(-g_5 ad\boldsymbol{X}_5)\boldsymbol{X}_5$, $\exp(-g_5 ad\boldsymbol{X}_5)\boldsymbol{X}_7$ nor $\exp(-g_5 ad\boldsymbol{X}_5)\boldsymbol{X}_8$ contribute to the coefficients of $\boldsymbol{X}_3$ or $\boldsymbol{X}_6$, and these elements do not therefore need to be considered. One then gets

$\boldsymbol{X}_1'(t) = \sin(-2g_2)\exp(-g_5 ad\boldsymbol{X}_5)\boldsymbol{X}_3 + \frac{1}{2}\sin(2g_2)\left(1 - \cos(2g_4)\right)\exp(-g_5 ad\boldsymbol{X}_5)\boldsymbol{X}_6$

Using

$\exp(-g_5 ad\boldsymbol{X}_5)\boldsymbol{X}_6 = (\cos(2g_5)\boldsymbol{X}_6 + \sin(2g_5)\boldsymbol{X}_4),$

$\exp(-g_5 ad\boldsymbol{X}_5)\boldsymbol{X}_3 = \boldsymbol{X}_3 + \frac{1}{2}\sin(2g_5)\boldsymbol{X}_4 + \frac{1}{2}\left(-1 + \cos(2g_5)\right)\boldsymbol{X}_6$

gives

$\boldsymbol{X}_1'(t) = \sin(-2g_2)\left(\boldsymbol{X}_3 + \frac{1}{2}\sin(2g_5)\boldsymbol{X}_4 + \frac{1}{2}\left(-1 + \cos(2g_5)\right)\boldsymbol{X}_6\right) + \frac{1}{2}\sin(2g_2)\big(1 -$

$\cos(2g_4)\big)(\cos(2g_5)\boldsymbol{X}_6 + \sin(2g_5)\boldsymbol{X}_4)$

The coefficients of $\boldsymbol{X}_4$ do not need to be considered, leading to

$\boldsymbol{X}_1'(t) = \sin(-2g_2)\boldsymbol{X}_3 + \frac{1}{2}\sin(2g_2)\left(1 - \cos(2g_4)\cos(2g_5)\right)\boldsymbol{X}_6$

In this way we get

$b_{13} = \sin(-2g_2)$

and

$b_{16} = \frac{1}{2}\sin(2g_2)\left(1 - \cos(2g_4)\cos(2g_5)\right)$

All other b_{n3} and b_{n6} can be found in this way. Hence, the requisite columns of **B** are calculated.Type equation here.

$$
\mathbf{B}(\mathrm{t}) = \begin{pmatrix}
b_{11} & b_{12} & \sin(-2g_2) & b_{14} & b_{15} & \frac{1}{2}\sin(2g_2)\big(1-\cos(2g_4)\cos(2g_5)\big) & b_{17} & b_{18} & b_{19} \\
b_{21} & b_{22} & \sin(2g_1)\cos(2g_2) & b_{24} & b_{25} & \frac{1}{2}\sin(2g_1)\cos(2g_2)(\cos(2g_4)\cos(2g_5)-1) & b_{27} & b_{28} & b_{29} \\
b_{31} & b_{32} & \cos(2g_1)\cos(2g_2) & b_{34} & b_{35} & \frac{1}{2}\cos(2g_1)\cos(2g_2)(\cos(2g_4)\cos(2g_5)-1) & b_{37} & b_{38} & b_{39} \\
b_{41} & b_{42} & 0 & b_{44} & b_{54} & b_{46} & b_{47} & b_{48} & b_{49} \\
b_{51} & b_{52} & 0 & b_{54} & b_{55} & b_{56} & b_{57} & b_{58} & b_{59} \\
b_{61} & b_{62} & \frac{1}{2}(\cos(2g_1)\cos(2g_2)-1) & b_{64} & b_{65} & b_{66} & b_{67} & b_{68} & b_{69} \\
b_{71} & b_{72} & 0 & b_{74} & b_{75} & b_{76} & b_{77} & b_{78} & b_{79} \\
b_{81} & b_{82} & 0 & b_{84} & b_{85} & b_{86} & b_{87} & b_{88} & b_{89} \\
b_{91} & b_{92} & \frac{1}{2}\big(1-\cos(2g_1)\cos(2g_2)\big) & b_{94} & b_{95} & b_{96} & b_{97} & b_{98} & b_{99}
\end{pmatrix}
$$

$$b_{46} = \big(\cos(g_1)\cos(g_2)\sin(g_3) - \sin(g_1)\sin(g_2)\cos(g_3)\big)\sin(2g_4)\cos(2g_5) - \big(\cos(g_1)\cos(g_2)\cos(g_3) + \sin(g_1)\sin(g_2)\sin(g_3)\big)\sin(2g_5)$$

$$b_{56} = \big(\sin(g_1)\sin(g_2)\sin(g_3) + \cos(g_1)\cos(g_2)\cos(g_3)\big)\sin(2g_4)\mathit{cos}(2g_5) - \big(\sin(g_1)\sin(g_2)\cos(g_3) - \cos(g_1)\cos(g_2)\sin(g_3)\big)\sin(2g_5)$$

$$b_{66} = \frac{1}{2}\frac{1}{2}(\cos(2g_1)\cos(2g_2)-1)(\cos(2g_4)\cos(2g_5)-1) + \cos(2g_4)\cos(2g_5)$$

$$b_{76} = \big(\mathit{cos}(g_1)\sin(g_2)\cos(g_3) + \sin(g_1)\cos(g_2)\sin(g_3)\big)\sin(2g_5) + \big(\sin(g_1)\cos(g_2)\cos(g_3) - \cos(g_1)\sin(g_2)\sin(g_3)\big)\sin(2g_4)\cos(2g_5)$$

$$b_{86} = \big(\sin(g_1)\cos(g_2)\cos(g_3) - \cos(g_1)\sin(g_2)\sin(g_3)\big)\sin(2g_5) - \big(\sin(g_1)\cos(g_2)\sin(g_3) + \cos(g_1)\sin(g_2)\cos(g_3)\big)\sin(2g_4)\cos(2g_5)$$

$$b_{86} = \frac{1}{2}\frac{1}{2}\big(-3+\cos(2g_1)\cos(2g_2)\big)\big(1-\cos(2g_4)\cos(2g_5)\big)$$

$$a_{16} = \frac{1}{2}(1-\cos(2g_4)\cos(2g_5))\sin(2g_2)$$

Section 3.4 Elements of the time correlation matrix of the dynamical symmetries, A.

We give in this section the elements of the first row of the time correlation matrix **A** which governs the time dependence of the dynamical symmetries, $\boldsymbol{\mathcal{X}}_k(t)$, for the 3 state model discussed in the main text

$$\boldsymbol{\mathcal{X}}_k(t) = \mathbf{U}\boldsymbol{X}_k\mathbf{U}^{-1} = \left(\prod_{j=1}^{\nu} \exp\big(g_j(t) ad\boldsymbol{X}_j\big)\right)\boldsymbol{X}_k = \sum_{m=1}^{\nu} a_{km}\boldsymbol{X}_m$$

$$\begin{aligned}
a_{11} = \sin(2g_2)\,(&\cos(g_4)\sin(g_4)(\cos(g_6)\sin(g_6)(2\cos(g_7)\cos(g_9)\sin(g_8)\\
&+ 2\cos(g_8)\sin(g_7)\sin(g_9)) + \cos(2g_6)\,(\cos(g_8)\cos(g_9)\sin(g_7)\\
&- \cos(g_7)\sin(g_8)\sin(g_9)))\\
&+ \cos(2g_4)\,\sin(2g_5)\,\cos(2g_6)\,(\frac{1}{2}\cos(g_7)\cos(g_9)\sin(g_8)\\
&+ \frac{1}{2}\cos(g_8)\sin(g_7)\sin(g_9)) + \cos(g_6)\sin(g_6)(-\cos(g_8)\cos(g_9)\sin(g_7)\\
&+ \cos(g_7)\sin(g_8)\sin(g_9))))\\
&+ \cos(2g_2)(\cos(2g_3)(\sin(g_4)\sin(g_5)(\cos(g_6 - g_9)\,\sin(g_7)\sin(g_8)\\
&+ \cos(g_7)\cos(g_8)\sin\,(g_6 - g_9))\\
&+ \cos(g_4)\cos(g_5)(\cos(g_6)\cos(g_7)\cos(g_8)\cos(g_9)\\
&- \cos(g_9)\sin(g_6)\sin(g_7)\sin(g_8) + \cos(g_7)\cos(g_8)\sin(g_6)\sin(g_9)\\
&+ \cos(g_6)\sin(g_7)\sin(g_8)\sin(g_9)))\\
&+ \sin(2g_3)(\cos(g_6)(\cos(g_7)\cos(g_8)(\cos(g_9)\sin(g_4)\sin(g_5)\\
&+ \cos(g_4)\cos(g_5)\sin(g_9)) + \sin(g_7)\sin(g_8)(-\cos(g_4)\cos(g_5)\cos(g_9)\\
&+ \sin(g_4)\sin(g_5)\sin(g_9)))\\
&+ \sin(g_6)(\sin(g_4)\sin(g_5)(-\cos(g_9)\sin(g_7)\sin(g_8)\\
&+ \cos(g_7)\cos(g_8)\sin(g_9)) + \cos(g_4)\cos(g_5)(-\cos(g_7)\cos(g_8)\cos(g_9)\\
&- \sin(g_7)\sin(g_8)\sin(g_9)))))
\end{aligned}$$

$$a_{12} = \sin(2g_2)\,(\sin(2g_4)(\cos(2g_6)(-\frac{1}{2}\cos(g_7)\cos(g_9)\sin(g_8)$$
$$-\frac{1}{2}\cos(g_8)\sin(g_7)\sin(g_9)) + \cos(g_6)\sin(g_6)(\cos(g_8)\cos(g_9)\sin(g_7)$$
$$-\cos(g_7)\sin(g_8)\sin(g_9)))$$
$$+\cos(2g_4)\sin(2g5)(\cos(g_6)\sin(g_6)(\cos(g_7)\cos(g_9)\sin(g_8)$$
$$+\cos(g_8)\sin(g_7)\sin(g_9)) + \cos(2g_6)(0.5\cos(g_8)\cos(g_9)\sin(g_7)$$
$$-\frac{1}{2}\cos(g_7)\sin(g_8)\sin(g_9))))$$
$$+\cos(2g_2)(\sin(g_4)\sin(g5)(\cos(g_6 - g_9)\,\sin(2g_3)\sin(g_7)\sin(g_8)$$
$$+\cos(g_7)\cos(g_8)(\cos(g_9)\sin(2g_3)\sin(g_6)$$
$$-2\cos(g_3)\cos(g_6)\sin(g_3)\sin(g_9))$$
$$+\cos(2g_3)(\sin(g_6)(\cos(g_9)\sin(g_7)\sin(g_8) - \cos(g_7)\cos(g_8)\sin(g_9))$$
$$+\cos(g_6)(-\cos(g_7)\cos(g_8)\cos(g_9) - \sin(g_7)\sin(g_8)\sin(g_9))))$$
$$+\cos(g_4)\cos(g_5)(\cos(g_6)(\cos(2g_3 - g_9)\,\sin(g_7)\sin(g_8)$$
$$+\cos(g_7)\cos(g_8)\,\sin(2g_3 - g_9))$$
$$+\sin(g_6)(-2\cos(g_3)\cos(g_9)\sin(g_3)\sin(g_7)\sin(g_8)$$
$$+\cos(g_7)\cos(g_8)\sin(2g_3)\sin(g_9) + \cos(2g_3)(\cos(g_7)\cos(g_8)\cos(g_9)$$
$$+\sin(g_7)\sin(g_8)\sin(g_9)))))$$

$$a_{13} = -\sin(2g_2)$$

$$
\begin{aligned}
a_{14} = \frac{1}{2}(\cos(g_7)(-8\cos(g_2)\cos(g_4)\cos(g_6)\cos(g_8)\cos(g_9)\sin(g_2)\sin(g_4)\sin(g_6) \\
+ \cos(2g_2)(\cos(g_5)\cos(2g_3 - g_4 + g_6 + g_9) \\
+ \cos(g_5)\cos(2g_3 + g_4 + g_6 + g_9) + \cos(2g_3 - g_4 + g_6 + g_9)\sin(g_5) \\
- \cos(2g_3 + g_4 + g_6 + g_9)\sin(g_5))\sin(g_8) \\
- \cos(2g_6)\cos(g_8)\sin(2g_2)\sin(2g_4)\sin(g_9)) \\
+ \cos(2g_4)\sin(2g_2)\sin(2g_5)(\sin(2g_6)(-\cos(g_9)\sin(g_7)\sin(g_8) \\
+ \cos(g_7)\cos(g_8)\sin(g_9)) + \cos(2g_6)(-\cos(g_7)\cos(g_8)\cos(g_9) \\
- \sin(g_7)\sin(g_8)\sin(g_9))) \\
+ \sin(g_7)(\cos(2g_6)\cos(g_9)\sin(2g_2)\sin(2g_4)\sin(g_8) \\
- \cos(g_2)\cos(g_4)\cos(g_6)\sin(g_2)\sin(g_4)\sin(g_6)\sin(g_8)\sin(g_9) \\
+ \cos(2g_2)\cos(g_8)\big(\cos(g_5) + \sin(g_5)\big)\sin(2g_3 - g_4 + g_6 + g_9) \\
+ \cos(2g_2)\cos(g_8)\big(\cos(g_5) - \sin(g_5)\big)\sin(2g_3 + g_4 + g_6 + g_9)
\end{aligned}
$$

$$a_{15} = \frac{1}{2}(\cos(g_9)(-\cos(2g_4)\cos(g_7)\cos(g_8)\sin(2g_2)\sin(2g_5)\sin(2g_6)$$
$$+ \cos(2g_2)\cos(g_8)(\cos(g_3)\sin(g_3)(-4\cos(g_6)\sin(g_4)\sin(g_5)$$
$$+ 4\cos(g_4)\cos(g_5)\sin(g_6)) + \cos(2g_3)(-2\cos(g_4)\cos(g_5)\cos(g_6)$$
$$- 2\sin(g_4)\sin(g_5)\sin(g_6)))\sin(g_7)$$
$$+ (\cos(2g_2)\cos(g_7)(\cos(2g_3)(-2\cos(g_6)\sin(g_4)\sin(g_5)$$
$$+ 2\cos(g_4)\cos(g_5)\sin(g_6))$$
$$+ \cos(g_3)\sin(g_3)(4\cos(g_4)\cos(g_5)\cos(g_6)$$
$$+ 4\sin(g_4)\sin(g_5)\sin(g_6)))$$
$$+ 8.\cos(g_2)\cos(g_4)\cos(g_6)\sin(g_2)\sin(g_4)\sin(g_6)\sin(g_7))\sin(g_8))$$
$$+ (-8\cos(g_2)\cos(g_4)\cos(g_6)\cos(g_7)\cos(g_8)\sin(g_2)\sin(g_4)\sin(g_6)$$
$$+ \cos(2g_2)\cos(g_8)(\cos(2g_3)(-2\cos(g_6)\sin(g_4)\sin(g_5)$$
$$+ 2\cos(g_4)\cos(g_5)\sin(g_6))$$
$$+ \cos(g_3)\sin(g_3)(4\cos(g_4)\cos(g_5)\cos(g_6)$$
$$+ 4\sin(g_4)\sin(g_5)\sin(g_6)))\sin(g_7)$$
$$+ (\cos(2g_2)\cos(g_7)(\cos(g_3)\sin(g_3)(4\cos(g_6)\sin(g_4)\sin(g_5)$$
$$- 4\cos(g_4)\cos(g_5)\sin(g_6)) + \cos(2g_3)(2\cos(g_4)\cos(g_5)\cos(g_6)$$
$$+ 2\sin(g_4)\sin(g_5)\sin(g_6)))$$
$$- \cos(2g_4)\sin(2g_2)\sin(2g_5)\sin(2g_6)\sin(g_7))\sin(g_8))\sin(g_9)$$
$$+ \cos(2g_6)\sin(2g_2)(\sin(g_7)\sin(g_8)(\cos(2g_4)\cos(g_9)\sin(2g_5)$$
$$+ \sin(2g_4)\sin(g_9)) + \cos(g_7)\cos(g_8)(\cos(g_9)\sin(2g_4)$$
$$- \cos(2g_4)\sin(2g_5)\sin(g_9))))$$

$$a_{16} = \frac{1}{2}(1 - \cos(2g_4)\cos(2g_5))\sin(2g_2)$$

$$a_{17}=\frac{1}{2}(3-\cos(2g_4)\cos(2g_5))\sin(2g_2)(\cos(2g_7)\cos(2g_9)\sin(2g_8)$$
$$+\sin(2g_7)\sin(2g_9))$$
$$+\cos(2g_2)(\cos(g_4)\sin(g_5)(\sin(2g_3)(\cos(2g_8)\cos(2g_9)\sin(g_6)$$
$$-\cos(g_6)\cos(2g_9)\sin(2g_7)\sin(2g_8)+\cos(g_6)\cos(2g_7)\sin(2g_9))$$
$$+\cos(2g_3)(\cos(2g_9)(\cos(g_6)\cos(2g_8)+\sin(g_6)\sin(2g_7)\sin(2g_8))$$
$$-\cos(2g_7)\sin(g_6)\sin(2g_9)))$$
$$+\cos(g_5)\sin(g_4)(\cos(g_6)(-\cos(2g_8)\cos(2g_9)\sin(2g_3)$$
$$+\cos(2g_3)(\cos(2g_7)\sin(2g_9)-\cos(2g_9)\sin(2g_7)\sin(2g_8)))$$
$$+\sin(g_6)(\cos(2g_3)\cos(2g_8)\cos(2g_9)$$
$$+\sin(2g_3)(-\cos(2g_9)\sin(2g_7)\sin(2g_8)+\cos(2g_7)\sin(2g_9)))))$$

$$a_{18}=\frac{1}{4}\sin(2g_2)((\cos(2g_4)\cos(2g_5)-3)\cos(2g_9)\sin(2g_7)+\frac{1}{4}(3$$
$$-\cos(2g_4)\cos(2g_5))\cos(2g_7)\sin(2g_8)\sin(2g_9))$$
$$+\cos(2g_2)(\sin(2g_3)(\cos(g_5)\sin(g_4)(-\cos(2g_7)\cos(2g_9)\sin(g_6)$$
$$-8\cos(g_7)\cos(g_8)\cos(g_9)\sin(g_6)\sin(g_7)\sin(g_8)\sin(g_9)$$
$$-\cos(g_6)\cos(2g_8)\sin(2g_9))$$
$$+\cos(g_4)\sin(g_5)(-\cos(g_6)\cos(2g_7)\cos(2g_9)$$
$$-8\cos(g_6)\cos(g_7)\cos(g_8)\cos(g_9)\sin(g_7)\sin(g_8)\sin(g_9)$$
$$+\cos(2g_8)\sin(g_6)\sin(2g_9)))$$
$$+\cos(2g_3)(\cos(g_5)\sin(g_4)(-\cos(g_6)\cos(2g_7)\cos(2g_9)$$
$$-8\cos(g_6)\cos(g_7)\cos(g_8)\cos(g_9)\sin(g_7)\sin(g_8)\sin(g_9)$$
$$+\cos(2g_8)\sin(g_6)\sin(2g_9))+\cos(g_4)\sin(g_5)(\cos(2g_7)\cos(2g_9)\sin(g_6)$$
$$+(\cos(g_6)\cos(2g_8)+\sin(g_6)\sin(2g_7)\sin(2g_8))\sin(2g_9))))$$

$$a_{19} = \frac{1}{4}(\cos(2g_4)\cos(2g_5) - 3)(1 - \cos(2g_7)\cos(2g_8))\sin(2g_2)$$
$$+ \cos(2g_2)(\cos(2g_3)(\cos(g_4)\sin(g_5)(\cos(2g_8)\sin(g_6)\sin(2g_7)$$
$$- 2\cos(g_6)\cos(g_8)\sin(g_8))$$
$$+ \cos(g_5)\sin(g_4)(-\cos(g_6)\cos(2g_8)\sin(2g_7)$$
$$- 2\cos(g_8)\sin(g_6)\sin(g_8)))$$
$$+ \sin(2g_3)(\cos(g_5)\sin(g_4)(-\cos(2g_8)\sin(g_6)\sin(2g_7)$$
$$+ 2\cos(g_6)\cos(g_8)\sin(g_8))$$
$$+ \cos(g_4)\sin(g_5)(-\cos(g_6)\cos(2g_8)\sin(2g_7)$$
$$- 2\cos(g_8)\sin(g_6)\sin(g_8)))$$

Reference

1. Wei, J.; Norman, E. Lie Algebraic Solution of Linear Differential Equations. *J. Math. Phys.* **1963**, *4*, 575-581, doi:10.1063/1.1703993.
2. Wei, J.; Norman, E. On Global Representations of the Solutions of Linear Differential Equations as a Product of Exponentials. *Proc. Am. Math. So.* **1964**, *15*, 327-334, doi:10.2307/2034065.
3. Altafini, C. Explicit Wei-Norman formulae for matrix Lie groups. In Proceedings of the Proc 41st IEEE Conf. on Decision and Control, 10-13 Dec. 2002, 2002; pp. 2714-2719
4. Altafini, C. Parameter differentiation and quantum state decomposition for time varying Schrödiner equations. *Reports on Mathematical Physics* **2003**, *52*, 381-400.
5. Remacle, F.; Levine, R.D. A quantum information processing machine for computing by observables. *Proc. Natl. Acad. Sci. USA* **2023**, *120*, e2220069120, doi:10.1073/pnas.2220069120.
6. Collini, E.; Gattuso, H.; Bolzonello, L.; Casotto, A.; Volpato, A.; Dibenedetto, C.N.; Fanizza, E.; Striccoli, M.; Remacle, F. Quantum Phenomena in Nanomaterials: Coherent Superpositions of Fine Structure States in CdSe Nanocrystals at Room Temperature. *The Journal of Physical Chemistry C* **2019**, *123*, 31286-31293, doi:10.1021/acs.jpcc.9b11153.
7. Gattuso, H.; Fresch, B.; Levine, R.D.; Remacle, F. Coherent Exciton Dynamics in Ensembles of Size-Dispersed CdSe Quantum Dot Dimers Probed via Ultrafast Spectroscopy: A Quantum Computational Study. *Applied Sciences* **2020**, *10*, 1328, doi:10.3390/app10041328.
8. Collini, E.; Gattuso, H.; Kolodny, Y.; Bolzonello, L.; Volpato, A.; Fridman, H.T.; Yochelis, S.; Mor, M.; Dehnel, J.; Lifshitz, E.; et al. Room-Temperature Inter-Dot Coherent Dynamics in Multilayer Quantum Dot Materials. *The Journal of Physical Chemistry C* **2020**, *124*, 1622-16231, doi:10.1021/acs.jpcc.0c05572.
9. Collini, E.; Gattuso, H.; Levine, R.D.; Remacle, F. Ultrafast fs coherent excitonic dynamics in CdSe quantum dots assemblies addressed and probed by 2D electronic spectroscopy. *Journal of Chemical Physics* **2021**, *154*, 014301, doi:10.1063/5.0031420.

10. Hamilton, J.R.; Amarotti, E.; Dibenedetto, C.N.; Striccoli, M.; Levine, R.D.; Collini, E.; Remacle, F. Harvesting a Wide Spectral Range of Electronic Coherences with Disordered Quasi-Homo Dimeric Assemblies at Room Temperature. *Advanced Quantum Technologies* **2022**, *5*, 2200060, doi:https://doi.org/10.1002/qute.202200060.
11. Hamilton, J.R.; Amarotti, E.; Dibenedetto, C.N.; Striccoli, M.; Levine, R.D.; Collini, E.; Remacle, F. Time-Frequency Signatures of Electronic Coherence of Colloidal CdSe Quantum Dot Dimer Assemblies Probed at Room Temperature by Two-Dimensional Electronic Spectroscopy. *Nanomaterials* **2023**, *13*, 2096, doi:10.3390/nano13142096.
12. Efros, A.L.; Rosen, M.; Kuno, M.; Nirmal, M.; Norris, D.J.; Bawendi, M. Band-edge exciton in quantum dots of semiconductors with a degenerate valence band: Dark and bright exciton states. *Physical Review B* **1996**, *54*, 4843-4856, doi:10.1103/PhysRevB.54.4843.
13. Norris, D.J.; Bawendi, M.G. Measurement and assignment of the size-dependent optical spectrum in CdSe quantum dots. *Physical Review B* **1996**, *53*, 16338-16346, doi:10.1103/PhysRevB.53.16338.
